# Loss of muscleblind-like 1 results in cardiac pathology and persistence of embryonic splice isoforms

**DOI:** 10.1038/srep09042

**Published:** 2015-03-12

**Authors:** Donald M. Dixon, Jongkyu Choi, Ayea El-Ghazali, Sun Young Park, Kenneth P. Roos, Maria C. Jordan, Michael C. Fishbein, Lucio Comai, Sita Reddy

**Affiliations:** 1 Department of Biochemistry and Molecular Biology, University of Southern California, Los Angeles, CA 90033, USA; 2 Department of Physiology, David Geffen School of Medicine at UCLA, Los Angeles, CA 90095, USA; 3 Department of Pathology and Laboratory Medicine, David Geffen School of Medicine at UCLA, Los Angeles, CA 90095, USA; 4 Department of Microbiology and Immunology, University of Southern California, Los Angeles, CA 90033, USA

## Abstract

Cardiac dysfunction is a prominent cause of mortality in myotonic dystrophy I (DM1), a disease where expanded CUG repeats bind and disable the muscleblind-like family of splice regulators. Deletion of muscleblind-like 1 (*Mbnl1^ΔE2/ΔE2^*) in 129 sv mice results in QRS, QTc widening, bundle block and STc narrowing at 2–4 months of age. With time, cardiac function deteriorates further and at 6 months, decreased R wave amplitudes, sinus node dysfunction, cardiac hypertrophy, interstitial fibrosis, multi-focal myocardial fiber death and calcification manifest. Sudden death, where no end point illness is overt, is observed at a median age of 6.5 and 4.8 months in ~67% and ~86% of male and female *Mbnl1^ΔE2/ΔE2^* mice, respectively. Mbnl1 depletion results in the persistence of embryonic splice isoforms in a network of cardiac RNAs, some of which have been previously implicated in DM1, regulating sodium and calcium currents, *Scn5a*, *Junctin*, *Junctate*, *Atp2a1*, *Atp11a*, *Cacna1s*, *Ryr2*, intra and inter cellular transport, *Clta*, *Stx2*, *Tjp1*, cell survival, *Capn3*, *Sirt2*, *Csda*, sarcomere and cytoskeleton organization and function, *Trim55*, *Mapt*, *Pdlim3*, *Pdlim5*, *Sorbs1*, *Sorbs2*, *Fhod1*, *Spag9* and structural components of the sarcomere, *Myom1*, *Tnnt2*, *Zasp*. Thus this study supports a key role for Mbnl1 loss in the initiation of DM1 cardiac disease.

Myotonic dystrophy type I (DM1) is a multi-system disorder occurring with an incidence of 1:8000 worldwide. Three broad forms of DM1 are described, a classic form which has an onset between 10 and 60 years, presenting with myotonia, muscle weakness, smooth and cardiac muscle involvement, CNS dysfunction, somnolence, endocrine disorders and cataracts, a congenital form which is symptomatic at birth and which progresses to manifest many of the symptoms of the classic form of the disease and a minimal form that has its onset after 50 years manifesting with cataracts, myotonia and mild muscle weakness^1^,^2^. Heart disease is a well-established feature of the classic and congenital forms of the disease, with cardiac sudden death a prominent cause of mortality^1^,^2^. Conduction disorders predominate in DM1. First degree heart block or PR prolongation is commonly observed in DM1 and with increasing time or enhanced disease severity, second and third degree heart block, QRS, QTc widening and bundle blocks increase in frequency^1^,^2^,^3^,^4^,^5^. Poor R wave progression is also a feature noted in DM1^6^. Left ventricular hypertrophy, dilation and systole dysfunction occur less frequently and can manifest in the absence conduction disorders^1^,^2^,^7^,^8^,^9^,^10^. Histopathological changes include multi focal myofibrillar loss, fatty infiltration and fibrosis^1^,^2^,^6^,^10^. A combination of these features is widely believed to contribute to sudden cardiac death^9^,^10^,^11^. Of the electrocardiograph measurements the sum of the QRS duration and PR interval has been shown to be a strong predictor of mortality in DM1^12^.

The genetic mutation in DM1 is the expansion of a CTG repeat sequence located in the 3′ untranslated region of *DMPK* and immediately 5′ of *SIX5* on chromosome 19q13.3^13^,^14^. In general, larger CTG expansions are associated with a higher incidence and progression of conduction disease and a trend towards an increase in serious cardiac events^15^. Several lines of evidence demonstrate that expression of expanded CUG repeat sequences plays an important role in the development of key aspects of DM1 pathology^16^,^17^,^18^,^19^. Toxicity associated with expanded CUG repeats, stems in part from its ability to aberrantly sequester and disable the muscleblind-like (MBNL) family of RNA splice regulators^20^,^21^,^22^. In humans, *MBNL1* is highly expressed in skeletal muscle and the heart and shows lower expression in the brain, lung, liver, kidney and pancreas^21^. In mice, although *Mbnl1* expression is more consistent across tissues, the highest expression of *Mbnl1* is observed in the heart^23^. Previous experiments have shown that Mbnl1 loss in mice results in skeletal muscle myotonia and histopathology characteristic of DM1, in conjunction with cataracts and behavioral alterations that are reminiscent of DM1 patients^24^,^25^. Mbnl1 is known to play an important role in regulating the transition of its target RNAs from the embryonic splice program to that of the adult^26^,^27^. Significantly, previous studies have implicated the persistence of embryonic *Clcn1* splice isoforms in adult muscle with the development of myotonia both in Mbnl1 deficient mice and in the *HSA^LR^* DM1 mouse model, where expanded CUG repeat RNA is expressed in skeletal muscle^24^,^28^. As morpholino antisense oligonucleotide targeting prevents aberrant *Clcn1* splicing and reverses myotonia in the *HSA^LR^* mice, a causal relationship is established between abnormal *Clcn1* splicing and myotonia^29^. Taken together these experiments highlight the potential of physiologically relevant Mbnl1 target RNAs that are aberrantly spliced to initiate DM1 pathology.

To test the role of Mbnl1 depletion in the development of DM1 cardiac pathology, we deleted *Mbnl1* exon 2 (*Mbnl1^ΔE2/ΔE2^*), which encodes the ATG codon, in 129 sv mice. This mutation results in the absence of the Mbnl1 protein. *Mbnl1^ΔE2/ΔE2^* mice show a shortened life span in conjunction with a variety of conduction defects, cardiac hypertrophy, fibrosis and multi-focal myocardial fiber death and calcification. Mbnl1 loss results in the enhanced expression of embryonic splice isoforms in an RNA network which regulates sodium and calcium currents, intra and inter cellular transport, cell survival, sarcomere and cytoskeleton organization and function and encoding structural components of the sarcomere. These results therefore support an important role for Mbnl1 depletion in the development of DM1 cardiac disease and suggest a role for altered splicing in initiating cardiac pathology.

## Results

### Development of *Mbnl1^ΔE2/ΔE2^* mice

**Figure 1:**
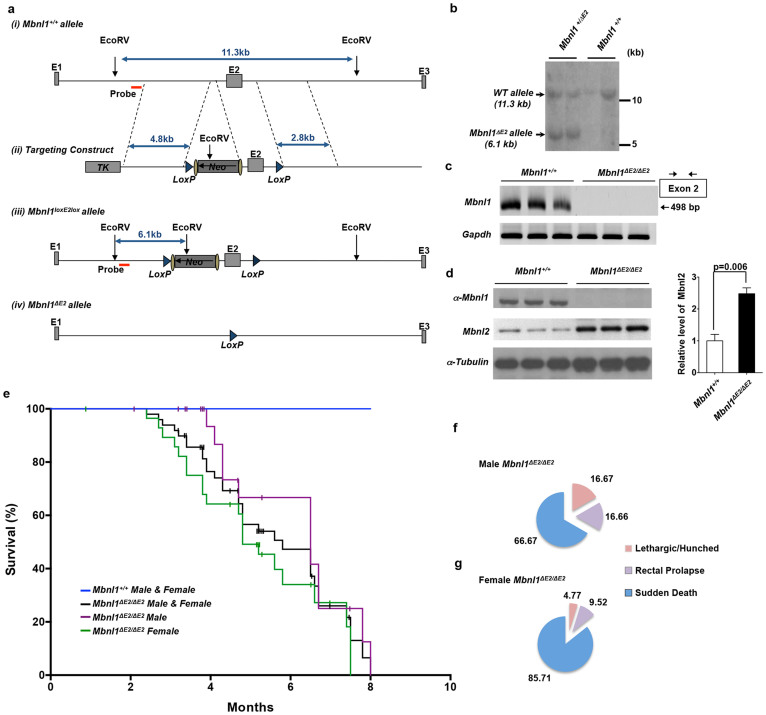
*Mbnl1^ΔE2/ΔE2^* mice demonstrate a reduced life-span. (a). The wild type (*Mbnl1^+/+^*) allele (i), targeting construct (ii), *Mbnl1^loxE2lox^* allele (iii) and *Mbnl1^ΔE2^* allele (iv) are shown. (b). Southern blot analysis of wild-type and targeted 129 sv ES cell DNA digested with EcoRV and analyzed with a probe indicted in Panel a. The image shown is cropped. The full-length gel is shown in Supplementary figure S1a. (c). RT-PCR analysis of *Mbnl1^+/+^* and *Mbnl1^ΔE2/ΔE2^* heart RNA using primers located in *Mbnl1* exon 2. *Gapdh* was amplified in parallel as an internal control. The images shown are cropped and full-length gels are shown in Supplementary figures S1b and c. (d). Western blot analysis of *Mbnl1^+/+^* and *Mbnl1^ΔE2/ΔE2^* heart protein lysates using anti-Mbnl1, anti-Mbnl2 and anti-α-tubulin antibodies. The images shown are cropped and the full-length gels are shown in supplementary figures S1d–f. (e). Kaplan-Meyer survival curves are shown. A total of 51 *Mbnl1^ΔE2/ΔE2^* mice (22 males, 29 females) and a corresponding number of male and female wild-type 129 sv mice were used in this study. Sacrificed mice are indicated by vertical bars. There is a statistically significant difference in the survival curves between *Mbnl1^ΔE2/ΔE2^* and *Mbnl1^+/+^* mice (χ^2^_(1)_ = 85.7, p < 0.00001). The survival curves between *Mbnl1^ΔE2/ΔE2^* males and *Mbnl1^ΔE2/ΔE2^* females is not statistically significant (χ^2^_(1)_ = 2.8, p < 0.10). (f, g). The recorded causes of death for the male (f) and female (g) *Mbnl1^ΔE2/ΔE2^* mice are indicated.

To test the role of MBNL1 deficiency in the development of DM1 cardiac disease we developed *Mbnl1^loxE2lox^* mice in which *Mbnl1* exon 2 was flanked by lox sites (Fig. 1a i–iii). Southern blot analysis of targeted 129 sv ES cells is shown in Fig. 1b. Chimeric animals derived from targeted 129 sv ES cells were bred to 129 sv wild type animals to derive *Mbnl1^loxE2lox^* mice (Fig. 1a iii). Lox mediated deletion of *Mbnl1* exon 2 was achieved by crossing *Mbnl1^loxE2lox^* mice with 129 sv transgenic mice expressing the Cre recombinase under the control of the protamine 1 promoter^30^. As the protamine 1 promoter drives expression of the Cre recombinase only in the male germ line, a cross between male *Mbnl1^loxE2lox/Cre^* mice and 129 sv wild-type females resulted in *Mbnl1^+/ΔE2^* mice, which were subsequently used to obtain *Mbnl1^ΔE2/ΔE2^* animals using standard breeding schemes (Fig. 1a iv). Deletion of *Mbnl1* exon 2 was established by RT-PCR analyses (Fig. 1c). Loss of Mbnl1 protein in *Mbnl1^ΔE2/ΔE2^* mice is shown by western blot analysis (Fig. 1d). Depletion of Mbnl1 resulted in ~2.5 fold increase in the steady-state levels of Mbnl2, a splice regulator, which is homologous to Mbnl1 (Fig. 1d). Analysis of genotype ratios of the progeny of male and female 129 sv *Mbnl1^+/ΔE2^* mice did not reveal a homozygous mutant lethal phenotype.

### *Mbnl1^ΔE2/ΔE2^* mice show a short life span

Kaplan-Meyer curves were developed to examine differences in survival between wild-type (*Mbnl1^+/+^*) and *Mbnl1^ΔE2/ΔE2^* animals. For this analysis 102 gender matched mice [*Mbnl1^+/+^*:51 and *Mbnl1^ΔE2/ΔE2^*: 51; for each genotype female n = 29 and male n = 22] were followed for up to 14.9 months (Fig. 1e–g). There was a statistically significant difference in the survival curves between *Mbnl1^ΔE2/ΔE2^* and *Mbnl1^+/+^* mice (χ^2^_(1)_ = 85.7, p < 0.00001). Median survival time was 6.5 months for *Mbnl1^ΔE2/ΔE2^* males (95% CI 6.48–6.59 months) and 4.8 months for *Mbnl1^ΔE2/ΔE2^* females (95% CI 4.05–5.62 months). The survival curves between *Mbnl1^ΔE2/ΔE2^* males and *Mbnl1^ΔE2/ΔE2^* females was not statistically significant (χ^2^_(1)_ = 2.8, p < 0.10). Death, where no end point symptoms or illness are overt, was observed in ~67% and ~86% of male and female *Mbnl1^ΔE2/ΔE2^* mice respectively. Rectal prolapse (male *Mbnl1^ΔE2/ΔE2^*: ~17% and female *Mbnl1^ΔE2/ΔE2^*: ~10%) or morbidity from an unknown etiology, where a hunched posture, weight loss and lethargy were observed prior to death (male *Mbnl1^ΔE2/ΔE2^*: ~17% and female *Mbnl1^ΔE2/ΔE2^*: ~5%) were the other prominent causes of mortality (Fig. 1f & g). No significant difference was observed between the body weights of male and female cohorts of *Mbnl1^+/+^* and *Mbnl1^ΔE2/ΔE2^* mice at 3–4 and 5–7 months of age (Supplementary Fig. S2).

### *Mbnl1^ΔE2/ΔE2^*mice show QRS, QTc widening, bundle block and STc shortening at 2 and 4 months of age

**Figure 2:**
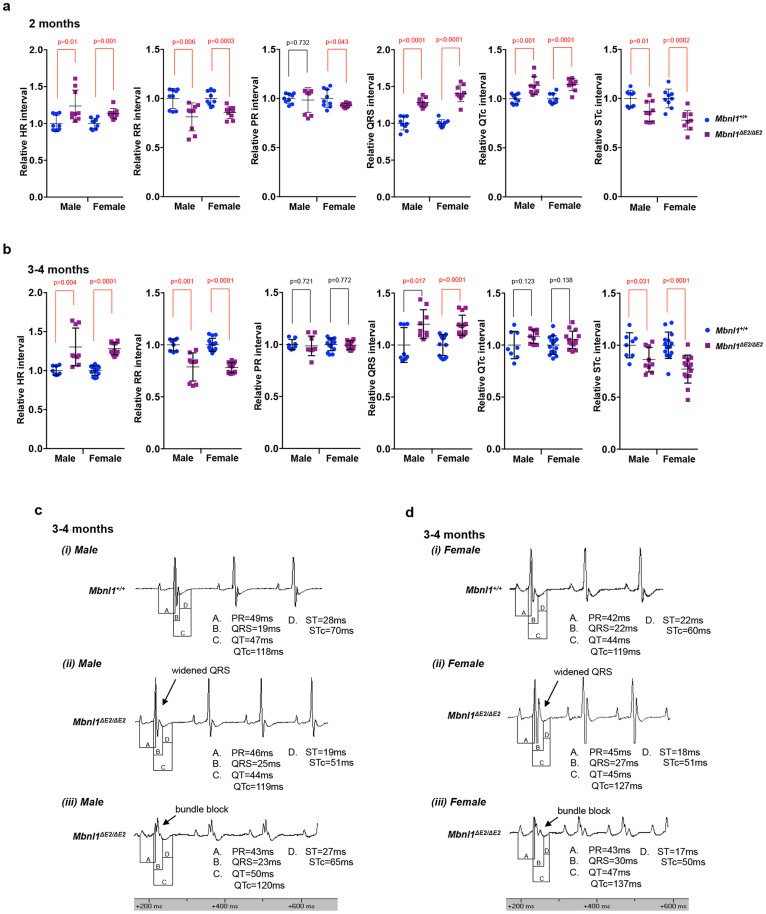
*Mbnl1^ΔE2/ΔE2^* mice show QRS, QTc widening, bundle block and STc shortening at 2 and 4 months of age. (a & b). Relative electrocardiogram interval values at 2 and 3–4 months of age for male (2 months: n = 3; 3–4 months: n = 3) and female (2 months: n = 3; 3–4 months: n = 5) *Mbnl1^+/+^* and male (2 months: n = 3; 3–4 months: n = 3) and female (2 months: n = 3; 3–4 months: n = 5) *Mbnl1^ΔE2/ΔE2^* mice are shown. Mice underwent light isoflurane anesthesia at an inducing dosage of 3% isoflurane in oxygen, which was reduced to 1–1.5% isoflurane in oxygen when measurements were recorded. As mouse ECG waveform shapes are different from human, QRS duration values in this study include the Tri (transient re-entry current) wave, as it is part of the ventricular depolarization phase. 80–125 beats were analyzed for each mouse. p-values were calculated using the Student's t-test with significance set at p ≤ 0.05. p values ≤0.05 are indicated in red. (c, d). Shown are representative electrocardiogram traces for male (c) and female (d) *Mbnl1^+/+^* and *Mbnl1^ΔE2/ΔE2^* mice at 3–4 months of age. QRS expansion (cii & dii) and bundle block (ciii & diii) are observed *Mbnl1^ΔE2/ΔE2^* mice. Bundle block was observed in ~30% of *Mbnl1^ΔE2/ΔE2^* mice in both age groups. Recorded values for all intervals are shown in Supplementary Fig. S3 & S4 and in Supplementary Tables S1 & S2.

As cardiac conduction defects are a prominent feature in DM1, surface electrocardiograms were recorded from male and female *Mbnl1^+/+^* and *Mbnl1^ΔE2/ΔE2^* mice at ~2 and 4 months of age under light isoflurane anesthesia. Male and female *Mbnl1^ΔE2/ΔE2^* mice showed a ~28% and ~40% increase in the QRS duration, respectively at 2 months (male: p < 0.0001; female: p < 0.0001) and a ~20% and ~19% increase in QRS duration at 4 months, respectively (male: p = 0.017; female: p < 0.0001) (Fig. 2a & b, c ii & d ii). Interestingly, ~30% of *Mbnl1^ΔE2/ΔE2^* mice studied at both ages showed bundle block, where the QRS complex shows an extra deflection, reflecting the different speeds with which depolarization occurs in the two ventricles (Fig. 2c iii & d iii). Bundle blocks were not observed in *Mbnl1^+/+^* animals. The QTc interval showed ~13% and ~14% increase in duration at 2 months of age in male and female *Mbnl1^ΔE2/ΔE2^* mice respectively (male: p = 0.001; female: p < 0.0001). A trend toward QTc widening was observed at 4 months of age in male and female *Mbnl1^ΔE2/ΔE2^* mice (Fig. 2a & b). The STc interval was ~13% and ~22% shorter in male and female *Mbnl1^ΔE2/ΔE2^* mice at 2 months of age (male: p = 0.01; female: p = 0.0002) and ~14% and ~23% shorter in male and female *Mbnl1^ΔE2/ΔE2^* mice at 4 months (male: p = 0.031; female: p < 0.0001) (Fig. 2a & b). Male and female *Mbnl1^ΔE2/ΔE2^* mice showed increased heart rates and shorter RR intervals (Fig. 2a & b). Data are mean and standard deviation. Recorded values are shown in Supplementary Fig. S3 & S4; Supplementary Table S1 & S2.

### Elongated QTc intervals, diminished R wave amplitudes and sinus node dysfunction occur in *Mbnl1^ΔE2/ΔE2^* mice at 6 months of age

**Figure 3:**
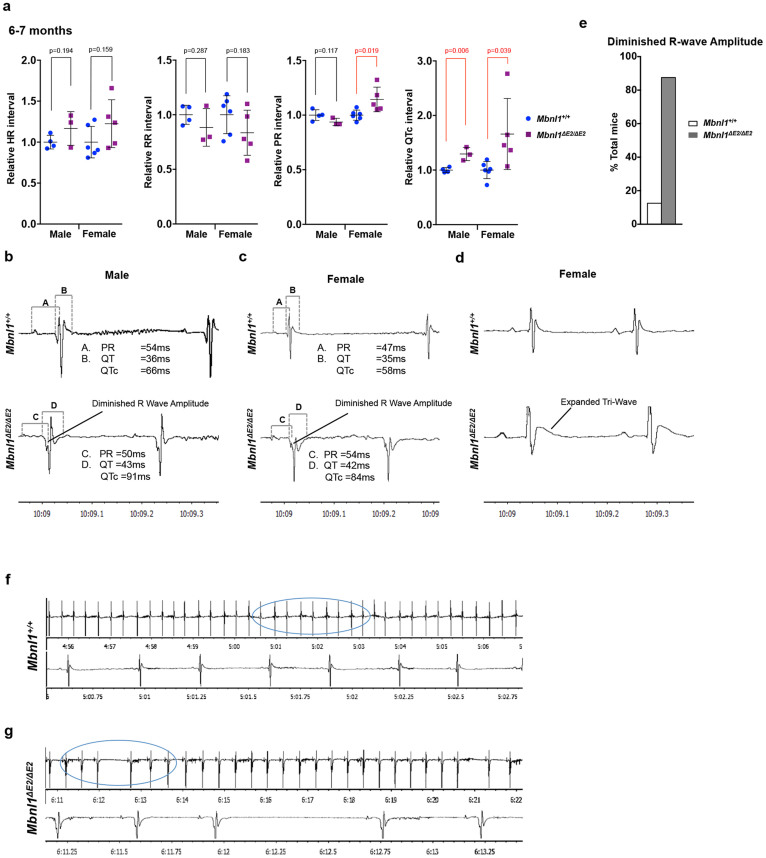
*Mbnl1^ΔE2/ΔE2^* mice anesthetized with ketamine/xylazine show prolonged QTc intervals, diminished R wave amplitudes and sinus node dysfunction at 6 months of age. (a). Relative electrocardiogram interval values for male (n = 4) and female (n = 6) *Mbnl1^+/+^* and male (n = 3) and female (n = 5) *Mbnl1^ΔE2/ΔE2^* mice at 6 months of age are shown. Mice underwent anesthesia via an intraperitoneal injection of 80 mg/kg ketamine and 8 mg/kg xylazine in saline. QRS durations include the Tri (transient re-entry current) wave, as it is part of the ventricular depolarization phase in mice. As S waves, following the triwaves, were not prominent in *Mbnl1^+/+^* and *Mbnl1^ΔE2/ΔE2^* mice after ketamine/xylazine treatment QRS intervals were not measured. Beats analyzed for each mouse were male (n = 74) and female (n = 77) *Mbnl1^+/+^* and male (n = 31) and female (n = 54) *Mbnl1^ΔE2/ΔE2^* mice. p-values were calculated using the Student's t-test with significance set at p ≤ 0.05. p-values ≤0.05 are indicated in red. (b–d). Representative electrocardiogram traces showing diminished R wave amplitudes in male (b) and female (c) *Mbnl1^ΔE2/ΔE2^* mice and expanded Tri-waves in female (d) *Mbnl1^ΔE2/ΔE2^* mice. (e): % *Mbnl1^+/+^* and *Mbnl1^ΔE2/ΔE2^* mice showing diminished R wave amplitudes. (f, g). Sporadic expansion of RR intervals to ≥200% of the average RR interval was observed in ~30% of the *Mbnl1^ΔE2/ΔE2^* mice analyzed [male (n = 4) and female (n = 6) *Mbnl1^+/+^* mice and male (n = 3) and female (n = 5) *Mbnl1^ΔE2/ΔE2^* mice]. Representative electrocardiogram traces from female *Mbnl1^+/+^* (f) and *Mbnl1^ΔE2/ΔE2^* (g) mice are shown. Recorded values for all intervals are shown in Supplementary Fig. S5 and in Supplementary Tables S3.

As *Mbnl1^ΔE2/ΔE2^* mice showed elevated heart rates, we reexamined the EKGs in male and female mice at 6 months of age subsequent to anesthesia with ketamine/xylazine, which reduces the heart rate and can therefore potentially uncover additional conduction defects. As predicted the heart rate was lower in both *Mbnl1^+/+^* and *Mbnl1^ΔE2/ΔE2^* mice with ketamine/xylazine administration when compared to that observed with isoflurane (Fig. 3a; Supplementary Table S3). As a prominent S wave was not observed with ketamine/xylazine, we measured QTc intervals. With ketamine/xylazine treatment, the QTc interval showed a prolongation of ~30% and ~66% in *Mbnl1^ΔE2/ΔE2^* male (p = 0.006) and female (p = 0.039) mice respectively (Fig. 3a–d). Data are mean and standard deviation. Recorded values are shown in Supplementary Fig. S5; Supplementary Table S3.

In these experiments we observed additional QRS waveform abnormalities including prolongation of the Tri wave (Transient reentry current) and sharply diminished R wave amplitudes (Fig. 3b–d). The prolonged Tri waves are consistent with QRS widening observed with isoflurane. Significantly, R wave amplitudes were strikingly smaller in ~90% of the *Mbnl1^ΔE2/ΔE2^* mice examined, with the R waves in most cases not reaching the isoelectric point (Fig. 3b, c & e). As poor R wave progression can reflect either lead misplacement, bundle blocks, prior myocardial infarcts, fibrosis or hypertrophy^31^ we repeated these experiments on a second EKG machine using a different type of acquisition system and leads and with an alternate investigator. As similar results were obtained in both cases, poor R wave amplitudes could reflect bundle blocks, myocardial fiber death, fibrosis or hypertrophy in *Mbnl1^ΔE2/ΔE2^* mice.

In ~25% of the *Mbnl1^ΔE2/ΔE2^* mice examined we observed elongated RR intervals (~1% to 2.5% of beats over a 20 minute interval), which reflected ≥200% increase in the time required for a new P wave to form. SA node firing was therefore inconsistent and sporadically required extended times to fire in *Mbnl1^ΔE2/ΔE2^* mice. Such events were not observed in *Mbnl1^+/+^* mice. A 5 minute interval EKG for a representative *Mbnl1^+/+^* mouse and an *Mbnl1^ΔE2/ΔE2^* mouse with elongated RR intervals is shown in Fig. 3f & g.

### *Mbnl1^ΔE2/ΔE2^* mice develop cardiac hypertrophy at 6 months of age

**Figure 4:**
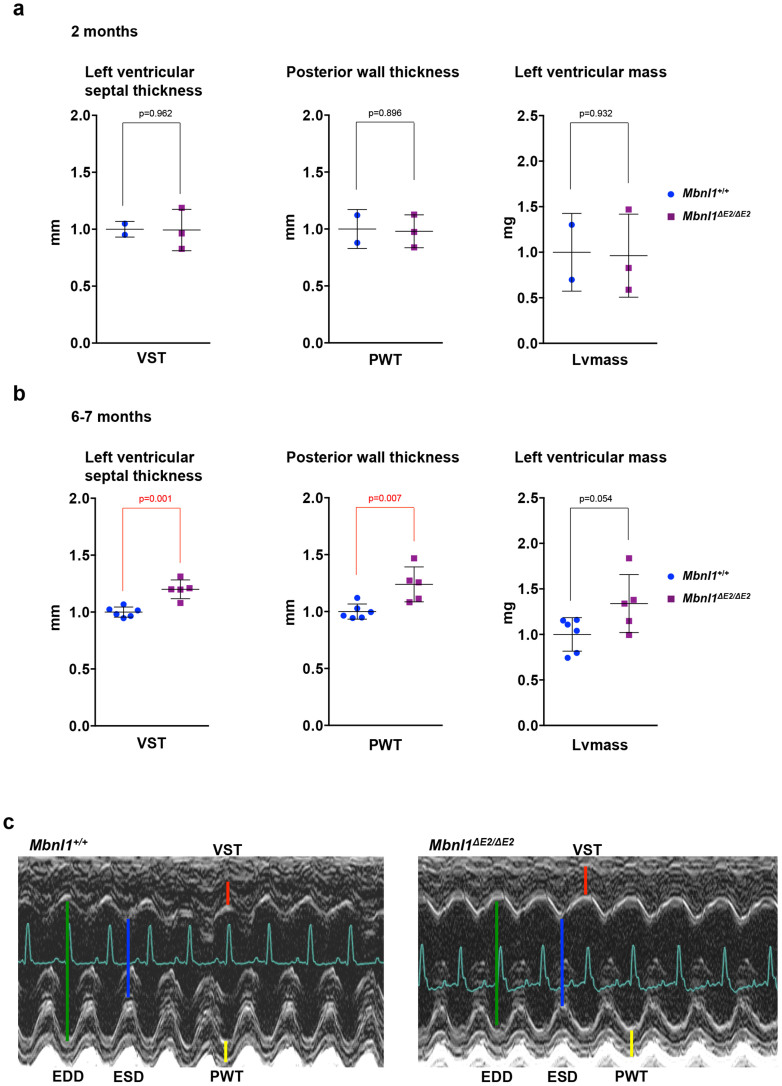
*Mbnl1^ΔE2/ΔE2^* mice show ventricular hypertrophy at 6 months of age. (a,b). Relative values for ultrasound echocardiographic measurements at 2 and 6–7 months of age for male *Mbnl1^+/+^* (2 months: n = 3; 6–7 months: n = 6) and *Mbnl1^ΔE2/ΔE2^* mice (2 months: n = 3; 6–7 months: n = 5) are shown. p-values were calculated using Student's t-test with significance set at p ≤ 0.05. Values where p ≤ 0.05 are shown in red. (c). 2-D guided M-Mode images of representative 20 week male *Mbnl1^+/+^* and *Mbnl1^ΔE2/ΔE2^* hearts. Ventricular septal thickness and posterior wall thickness are enlarged in the male *Mbnl1^ΔE2/ΔE2^* hearts. The depth from top to bottom is 7 mm. Time is 100 ms/div. Abbreviations: EDD: End-diastolic dimension (green). ESD: End-systolic dimension (blue). VST: Left ventricular septal thickness (red); PWT: Posterior wall thickness (yellow). Recorded values are shown in Supplementary Fig. S6 and in Supplementary Tables S4 & S5).

Left ventricular function and chamber dimensions were measured by ultrasound echocardiography at ~2 and 6 month of age in male and female *Mbnl1^+/+^* and *Mbnl1^ΔE2/ΔE2^* mice (Fig. 4; Supplementary Figure S6 and Supplementary Table S4 & S5). No abnormalities were noted at 2 months of age in male *Mbnl1^ΔE2/ΔE2^* mice (*Mbnl1^+/+^* n = 3; *Mbnl1^ΔE2/ΔE2^* n = 3) (Fig. 4a; Supplementary Table S4). Echocardiography could not be performed successfully on female mice at 2 months of age due to their small size. At 6 months of age, posterior wall thickness (PWT) was ~24% greater (p = 0.007), ventricular septal thickness (VST) ~20% greater (p = 0.001) and left ventricular mass ~34% greater (p = 0.054) in male *Mbnl1^ΔE2/ΔE2^* mice when compared to male *Mbnl1^+/+^* mice (*Mbnl1^+/+^* n = 6; *Mbnl1^ΔE2/ΔE2^* n = 5) (Fig. 4b & c; Supplementary Table S5). Left ventricular function was not diminished in male *Mbnl1^ΔE2/ΔE2^* mice. In females significant alterations in the left ventricular function and chamber dimensions were not observed at 6 months of age (*Mbnl1^+/+^* n = 3; *Mbnl1^ΔE2/ΔE2^* n = 5). However 2/5 female *Mbnl1^ΔE2/ΔE2^* mice tested at 6 months of age showed ~200–300% increase in left ventricle mass and ~30–60% decrease in left ventricle percent fractional shortening (Supplementary Table S5).

### Multi-focal calcification of myocardial fibers and interstitial fibrosis occur with Mbnl1 depletion at 6 months of age

**Figure 5:**
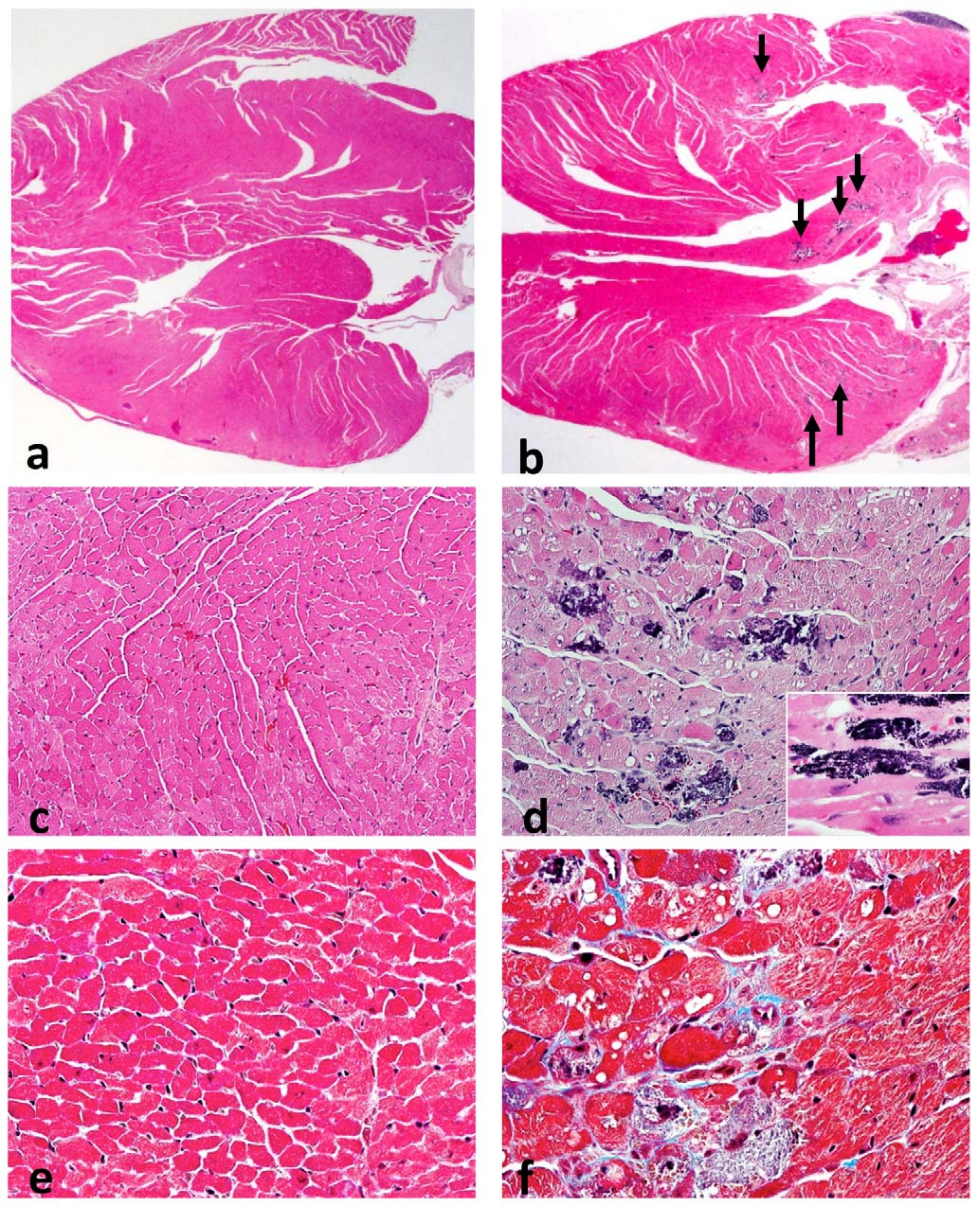
Multi-focal calcification and fibrosis in *Mbnl1^ΔE2/ΔE2^* hearts at 6 months of age. (a–f). Histological findings in 6 month female *Mbnl1^+/+^* (a, c, e) hearts (n = 3) and female *Mbnl1^ΔE2/ΔE2^* (b, d, f) hearts (n = 3) are shown. In *Mbnl1^+/+^* mice fibrosis and calcifications were not observed ((a): H&E stain ×12.5, (c): H&E stain ×200 and (e): trichrome stain ×400). In the *Mbnl1^ΔE2/ΔE2^* mice there are multiple foci of calcified myocardial fibers that appear dark blue in the H&E stained sections (arrows) ((b): H&E stain ×40, (d): H&E ×200). The inset shows individual blue dots that likely represent calcification of individual mitochondria (x400). In panel (f), a focus of calcification with interstitial fibrosis (light blue lines in this trichrome stained section, ×400) is shown. Both the calcification and the fibrosis were multifocal, involving less than 1% of the myocardial tissue, and were seen in all the *Mbnl1^ΔE2/ΔE2^* animals.

**Figure 6:**
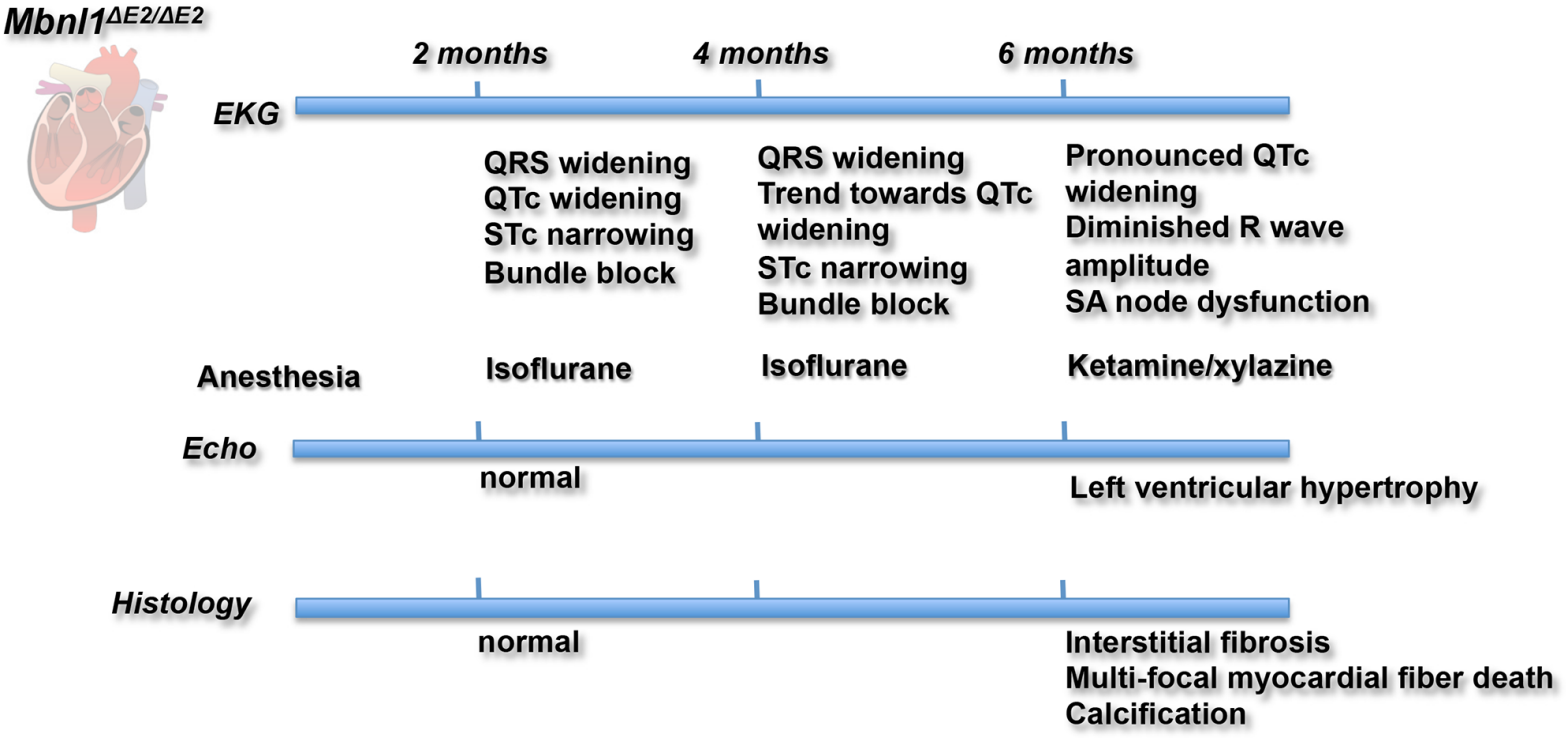
Cardiac pathology observed over 2–6 months of age in *Mbnl1^ΔE2/ΔE2^* mice. The heart image shown is obtained from ClipArtist.net (http://www.clipartlord.com/free-human-heart-clip-art/).

As poor R wave progression can reflect fibrosis or prior myocardial cell death, a histological analysis of *Mbnl1^+/+^* (n = 3) and *Mbnl1^ΔE2/ΔE2^* (n = 3) female hearts at 6 months of age was carried out using H&E and E trichrome stains. *Mbnl1^ΔE2/ΔE2^* heart sections showed multi-focal calcified myocardial fibers and interstitial fibrosis prominently in the interventricular septum and ventricles (Fig. 5). Such structural alterations can act as electrical insulators blocking or altering the course of the action potential depolarization wavefront and thus contributing to the conduction defects observed in *Mbnl1^ΔE2/ΔE2^* hearts at 6 months of age. Histological alterations were not observed at 2 months of age in *Mbnl1^ΔE2/ΔE2^* hearts (data not shown). Thus taken together these analyses demonstrate that Mbnl1 depletion results in progressive cardiac structural and functional abnormalities (Fig. 6).

### Increased embryonic isoform expression in *Mbnl1^ΔE2/ΔE2^* hearts

**Figure 7:**
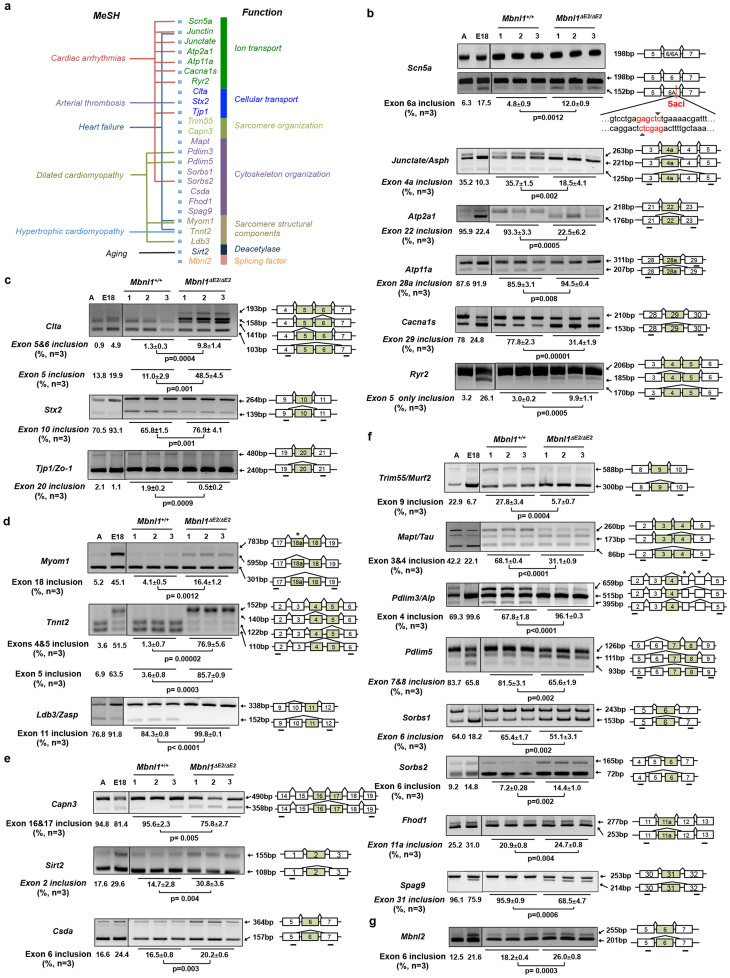
*Mbnl1* depletion results in the persistence of embryonic splice isoforms in adult hearts. (a). A cardiac RNA network that is misspliced in *Mbnl1^ΔE2/ΔE2^* hearts is shown. RNAs are categorized by their biological function and Mesh terms. (b–g). Splicing analysis for the indicated RNAs was performed by RT-PCR with E18 *Mbnl1^+/+^* and 4 months old male *Mbnl1^+/+^* (n = 3) and male *Mbnl1^ΔE2/ΔE2^* (n = 3) hearts. Band intensities were quantified by densitometry. Data are standard error of mean (SEM). p-values were calculated using Student's t-test with significance set at p ≤ 0.05. Primer locations, exon numbers and expected band sizes are indicated. The alternatively spliced exons are shown as green boxes. Exon numbers for all genes except *Junctate/Asph* are annotated based on Refseq from the UCSC genome browser (NCBI37/mm9); *Junctate/Asph* is annotated based on the study carried out by Dinchuck et al (Dinchuk et al., 2000). *Scn5a* exon 6 and 6A were distinguished by digestion with SacI. The SacI site within *Scn5* exon 6A is indicated in red. Identified novel exons are indicated by asterisks and the sequences of these exons are shown in Supplementary figure S9. The images shown are cropped. The full-length gels are shown in Supplementary figures S10–S15.

**Figure 8:**
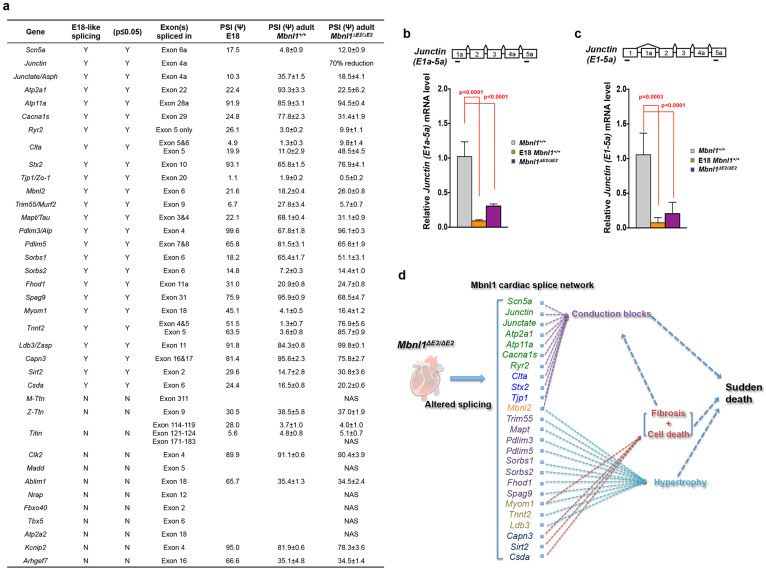
Tabulation of splice errors in *Mbnl1^ΔE2/ΔE2^* hearts. (a). Splice events examined in this study are tabulated. PSI: percent spliced in; NAS: Alternative splicing not detected by RT-PCR analysis. (b, c). Steady-state *Junctin (E1a–5a)* and *Junctin (E1–5a)* mRNA levels were analyzed by qPCR in E18 *Mbnl1^+/+^* hearts, 4 month male *Mbnl1^+/+^* (n = 3) and 4 month male *Mbnl1^ΔE2/ΔE2^* (n = 3) hearts. Exon numbers and primer positions are shown. Data are standard error of mean (SEM). p-values were calculated using Student's t-test with significance set at p ≤ 0.05. Values where p ≤ 0.05 are shown in red. (d). Potential relationships between splice errors and the cardiac pathology observed are indicated. The heart image shown is obtained from ClipArtist.net (http://www.clipartlord.com/free-human-heart-clip-art/).

As Mbnl1 is known to regulate RNA splice transitions during development, we curated a list of splice events in RNAs reported to be regulated by Mbnl1 in skeletal muscle and which are expressed at low levels in the heart^26^,^32^ from RNA-seq data obtained from a 129 sv/BL6 Mbnl1 knockout mouse developed by Kanadia and colleagues^24^, which was back crossed to wild-type 129 sv mice for more than 4 generations^27^ and splice events that are developmentally regulated in the heart^33^ as a reference (Fig. 7 & 8; Supplementary Fig. S7). As *Scn5a* exon 6a inclusion is developmentally regulated^33^ and because recent studies have implicated *Scn5a* exon 6a missplicing in the development of DM1 conduction defects and sudden death^34^ we studied this splice event and observed enhanced *Scn5a* exon 6a inclusion in adult *Mbnl1^ΔE2/ΔE2^* hearts in a manner reminiscent of DM1 and similar to that observed in E18 *Mbnl1^+/+^* hearts (Fig. 7b). Three independent RNAs, *Junctin*, *Junctate* and *Asph* are derived from a single genomic locus by alternative splicing^35^. *Asph* Exon 4a inclusion is diminished in E18 *Mbnl1^+/+^* hearts and in adult *Mbnl1^ΔE2/ΔE2^* hearts when compared with adult *Mbnl1^+/+^* hearts (Fig. 7b). *Asph* Exon 4a is present in one of four alternatively spliced *Asph* isoforms, one of two *Junctate* isoforms and in both alternatively spliced forms of the *Junctin* RNA in the mouse heart^35^. To confirm that the two alternatively spliced isoforms of junctin (*Asph* Exon1a–5a and *Asph* Exon 1–5a) show constitutive inclusion of exon 4a in 129 sv mice, we examined the two known *Junctin* isoforms by RT-PCR. A single band was observed when primers were located in *Asph* exon 1 and exon 5a and when primers were located in *Asph* exon 1a and exon 5a (Supplementary Fig. S8), demonstrating that exon 4a is included in both *Junctin* RNA isoforms. As *Asph* exon 4a inclusion is diminished in adult *Mbnl1^ΔE2/ΔE2^* hearts, this splice error is predicted to decrease *Junctin* levels. Steady-state levels of *Junctin (Exon 1a–5a)* and *Junctin (Exon 1–5a)* mRNA levels measured by qPCR were ~70% and ~80% reduced respectively, in *Mbnl1^ΔE2/ΔE2^* hearts when compared to controls (Fig. 8a–c; Supplementary Fig. S8). Diminished *Atp2a1* exon 22 inclusion, enhanced inclusion *Atp11a* exon 28a, diminished inclusion of *Cacna1s* exon 29 and enhanced *Ryr2* exon 5 only inclusion (Fig. 7b) are observed in *Mbnl1^ΔE2/ΔE2^* hearts and E18 *Mbnl1^+/+^* hearts when compared with adult *Mbnl1^+/+^* hearts. Thus RNAs regulating sodium and calcium currents show increased expression of embryonic splice isoforms in *Mbnl1^ΔE2/ΔE2^* hearts. With Mbnl1 depletion, three RNAs involved in inter and intra cellular transport *Clta, Stx2* and *Tjp1/Zo1* are aberrantly spliced in a manner reminiscent of E18 *Mbnl1^+/+^* hearts (Fig. 7c). *Atp2a2* exon 18 and *Kcnip2* exon 4 splicing was not altered in *Mbnl1^ΔE2/ΔE2^* hearts (Fig. 8; Supplementary Fig. S7).

We examined splice site choice in *Titin* (*Ttn*) and *Myomesin I* (*Myom1*) RNAs, which encode PEVK or PEVK-like domains that contribute to protein elasticity and can therefore regulate the compliance of the myocardium^36^,^37^,^38^,^39^,^40^. In addition, we examined splice events in *Capn3*, *Sirt2*, *Csda*, *Clk2* and *Madd*, which are reported to regulate cell viability^41^,^42^,^43^,^44^,^45^,^46^. We did not observe missplicing of *Ttn* exon 9, *Ttn* exon 311, or *Ttn* exons 114–119, 121–124 or 171–183 in *Mbnl1^ΔE2/ΔE2^* hearts (Supplementary Fig. S7). Mbnl1 loss resulted in enhanced inclusion of *Myom1*-exon 18 (Fig. 7d), diminished *Capn3* exon 16 and 17 inclusion, enhanced *Sirt2* exon 2 inclusion and enhanced *Csda* exon 6 inclusion (Fig. 7e). Mbnl1 depletion did not alter *Madd* exon 5 or *Clk2* exon 4 splicing (Fig. 8; Supplementary Fig. S7). A novel splicing event detected in *MyomI* is shown in Supplementary Fig. S9.

We tested splice events in additional RNAs encoding sarcomere proteins (*Tnnt2*, *Ldb3/Zasp*), proteins regulating sarcomere and cytoskeleton assembly and function (*Ablim1, Nrap*, *Mapt*, *Trim55/Murf2*, *Pdlim3*, *Pdlim5*, *Sorbs1*, *Sorbs2*, *Spag9*, *Fhod1* and *Arhgef7*). From this group of RNAs, *Tnnt2*, *Ldb3/Zasp*, *Trim55*, *Mapt*, *Pdlim3*, *Pdlim5*, *Sorbs1*, *Sorbs2*, *Fhod1* and *Spag9* RNAs are aberrantly spliced in *Mbnl1^ΔE2/ΔE2^* hearts, in a manner similar to that observed in E18 *Mbnl1^+/+^* hearts (Fig. 7 d & f). *Arhgef7* exon 16, *Nrap* exon 12 and *Ablim1* exon 18 splicing was not altered in *Mbnl1^ΔE2/ΔE2^* hearts (Fig. 8; Supplementary Fig. S7). Novel splice events detected in *Pdlim3/Alp* are shown in Supplementary Fig. S9.

Lastly, we examined splice events in RNAs known to play a role in heart development (*Tbx5*)^47^, myogenesis (*Fbxo40*)^48^ and splicing (*Mbnl2*)^22^. We observe enhanced inclusion of *Mbnl2* exon 6 (Fig. 7g), but splicing of *Tbx5* exon 6 and *Fbxo40* exon 2 was not altered in *Mbnl1^ΔE2/ΔE2^* hearts (Fig. 8; Supplementary Fig. S7). The RNA network where embryonic isoforms persist in adult *Mbnl1^ΔE2/ΔE2^* heart is shown in Fig. 7a.

## Discussion

Mbnl1 depletion in 129 sv mice results in QRS and QTc widening, STc shortening, bundle blocks, diminished R wave amplitudes and SA node dysfunction in conjunction with cardiac hypertrophy, fibrosis, multi-focal myofibrillar death, calcification and sudden death. Concurrently Mbnl1 depletion results in the enhanced expression of embryonic splice isoforms of RNAs regulating sodium and calcium currents (*Scn5a, Asph, Junctin, Junctate, Atp2a1, Atp11a, Cacna1s, Ryr2*), intra and inter cellular transport (*Clta, Stx2* and *Tjp1/Zo-1*), compliance of the myocardium (*Myom1*), cell survival (*Capn3, Sirt2, Csda*) cytoskeleton and sarcomere assembly and function (*Trim55*, *Mapt*, *Pdlim3*, *Pdlim5*, *Sorbs1*, *Sorbs2*, *Fhod1* and *Spag9*) and encoding sarcomere structural proteins (*Tnnt2, Ldb3*). As several of these features are observed in DM1^1^,^2^,^3^,^4^,^5^,^6^,^7^,^8^,^9^,^10^,^11^,^12^,^32^,^34^,^49^,^73^, this study supports a key role for Mbnl1 depletion in the initiation of DM1 cardiac pathology.

We examined the cardiac pathology that manifests over a period of two to six months in *Mbnl1^ΔE2/ΔE2^* mice and observe that QRS widening manifests at two months, is sustained at 4 months of age and that other distinctive QRS waveform abnormalities manifest at 6 months of age. Bundle block is observed in the 2–4 month period. Interestingly, QRS and QTc widening were more prominent at 2 months when compared to 4 months in *Mbnl1^ΔE2/ΔE2^* mice. This may reflect early mortality of the more severely affected animals (Fig.1e). Interestingly, as with DM1, where functional and structural involvement is associated with age and the male gender^10^,^15^, significant cardiac hypertrophy manifested at 6 months of age in male *Mbnl1^ΔE2/ΔE2^* mice, with females showing a trend towards hypertrophy. In contrast, QTc widening in DM1 is associated with being female and older^5^. Reminiscent of this feature, QTc widening was more prominent at 6 months of age in female *Mbnl1^ΔE2/ΔE2^* mice when compared to males. Cardiac histology in *Mbnl1^ΔE2/ΔE2^* mice showed similarities with DM1 with the manifestation of multi-focal calcification and fibrosis located primarily in the septum and ventricles at 6 months of age^10^. Such histological alterations could underlie the conduction defects observed at 6 months. The concurrent manifestation of conduction defects, ventricular hypertrophy and histological abnormalities may therefore serve to increase the predisposition to sudden cardiac death with Mbnl1 depletion. In this regard it is of interest to note that QRS, QTc widening and echocardiographic abnormalities have been implicated as risk factors for sudden death in DM1 patients^5^,^9^,^10^,^11^,^12^.

Our current and previous analysis of DM1 mouse models shows that DM1 cardiac pathology can result from DMPK, SIX5 and MBNL deficits^50^,^51^,^52^,^53^. CTG expansion is known to result in an ~20–75% decrease in DMPK^54^,^55^ and ~50% decrease in SIX5 levels^56^,^57^, as a consequence of *DMPK* RNA retention in the nucleus and epigenetic silencing of the *SIX5* allele linked to the expanded CTG tract. We have shown that first, second and third degree AV block manifest in mice as a function of time and diminishing Dmpk dosage ranging from 50–100% loss^50^,^51^,^52^. In other experiments we have shown that a *Six5+/−* mice show QRS widening^53^. As almost a complete sequestration of MBNL1 in CUG foci can occur in DM1 patient hearts with advanced disease^49^, we examined the consequence of complete Mbnl1 loss in the mouse heart and observe that Mbnl1 loss plays a key role in initiating the electro and echocardiographic abnormalities detailed above. In a recent study, Lee and colleagues have shown that combinatorial loss of Mbnl1 and Mbnl2 in 129 sv/BL6 mice (*Mbnl1−/−/Mbnl2+/−*) show both PR expansion or first degree heart block in conjunction with QRS expansion^58^. Taken together these results demonstrate that losses in DMPK, SIX5, MBNL1 and MBNL2 can recreate a great number of the prominent cardiac features observed in DM1. As systole dysfunction and diminished ejection fraction is not observed in these mouse models it is unclear if additional losses in Mbnl3 will be required to fully recapitulate both the echo and electrocardiographic abnormalities described in DM1. Significantly, CUG repeat expression also results in elevated steady state levels of the RNA binding protein, Cug-bp1^18^ and transgenic mice that acutely express Cug-bp1 levels at levels that are ~4–8 fold higher than endogenous levels show PR and QRS prolongation, dilated cardiomyopathy, systolic dysfunction with reduced ejection fractions and widespread necrosis with death occurring 7 days post induction of Cug-bp1 expression^59^. As the acute induction of high levels of CUG-BP1 is not observed in DM1 patients, it is important to examine the nature of the pathology that manifests with the constitutive expression of Cug-bp1 in mice, at levels that are similar to that observed in DM1 patient hearts. Such experiments will allow better comparisons to be made between the pathology resulting from CUG-BP1 over-expression and reduced levels of DMPK, SIX5 and the MBNL proteins. As Cug-bp1 over-expression alters splice site choice in a manner that partially overlaps with that observed with Mbnl1 loss^60^, pathophysiology shared by both mouse models has been hypothesized to result from similar RNA defects, while unique features and the disease trajectory of the cardiac pathology observed in *Mbnl1^ΔE2/ΔE2^* hearts are predicted to result from RNA defects resulting exclusively from Mbnl1 loss.

Lee et al.^58^ have demonstrated that *Mbnl1* exon 2 deleted mice developed by Kanadia and colleagues^24^ on a mixed 129 sv/BL6 background do not show cardiac pathology (annotated as *Mbnl1−/−* by the authors). These animals showed modest to severe splice errors in 8 RNAs. The lack of a cardiac phenotype was attributed to a compensatory increase in Mbnl2 steady-state levels in 129 sv/BL6 *Mbnl1−/−* hearts. Reduction in Mbnl2 levels in conjunction with Mbnl1 loss in 129 sv/BL6 *Mbnl1−/−/Mbnl2+/−* mice resulted in a decrease in life span and the development of cardiac pathology comparable to that described for the 129 sv *Mbnl1^ΔE2/ΔE2^* mice in this study. It should be noted, that both in this study and that of Lee and colleagues *Mbl1* exon 2, which encodes the Mbnl1 translational start site, was deleted. Furthermore, both 129 sv/BL6 *Mbnl1−/−* and 129 sv *Mbnl1^ΔE2/ΔE2^* hearts show an increase in steady-state Mbnl2 levels (Fig. 1d). However, when *Tnnt2* exon 5 inclusion, a splice error studied in all three mouse strains is examined interesting differences come to light. In 129 sv/BL6 *Mbnl1−/−* hearts, aberrant *Tnnt2* exon 5 inclusion is ~20%. In contrast both 129 sv/BL6 *Mbnl1−/−/Mbnl2+/−* hearts and 129 sv *Mbnl1^ΔE2/ΔE2^* hearts show ~70–85% inclusion of *Tnnt2* exon 5 (Fig. 7d). In contrast *Arhgef7* was misspliced in 129 sv/BL6 *Mbnl1−/−* hearts but not in 129 sv *Mbnl1^ΔE2/ΔE2^* hearts (Supplementary Fig. S7 and Ref. 58). These results demonstrate that mouse strain differences can alter the outcome of both Mbnl1 mediated splicing and cardiac pathology. This observation is significant, as DM1 is recognized to be a disorder characterized by significant phenotypic variability associated with CTG tract expansions of similar lengths^61^. Our results support the hypothesis that modifier genes regulating Mbnl1 function can strongly influence disease trajectory in DM1 patients. Thus comparison of these two mouse models provides a unique opportunity to identify modifiers that regulate the severity of DM1 pathology. Identification of such modifier genes will allow better predictions to be made for disease trajectories in individual patients and provide mechanistic insights into Mbnl1 regulation *in vivo*.

Splice events that we have tested in *Mbnl1^ΔE2/ΔE2^* hearts are shown in Fig. 7 & 8. As several lines of evidence point to alterations in myocyte force generation, force transmission via the cytoskeleton and calcium homeostasis as critical signals that result in cardiac remodeling and disease^62^, interesting correlations exist between the RNAs that show aberrant splicing and the *Mbnl1^ΔE2/ΔE2^* heart phenotype. A subset of splice events that are noteworthy with respect to the pathology observed in *Mbnl1^ΔE2/ΔE2^* hearts are discussed. A central pathology resulting from Mbnl1 loss is conduction defects, which can reflect both functional and structural cardiac abnormalities. With regard to functional deficits, *Scn5a* and *Junctin/Junctate* missplicing is of interest. Adult *Mbnl1^ΔE2/ΔE2^* hearts show ~2.5 fold increase in the inclusion of *Scn5a* exon 6a (~12%) (Fig. 7b). Inclusion of exon 6a in *Scn5a* results in a sodium current loss of function phenotype, which manifests with a depolarized shift in steady-state activation, slower kinetics of activation and inactivation, slower recovery from inactivation, an increase in the time for the sodium currents to peak and reduced channel availability^63^. This splice error has been recently reported in DM1 patients and is predicted to contribute to sudden death in this patient population^34^. As the QRS interval duration is influenced by the time for sodium currents to peak^64^, enhanced inclusion of Scn5a exon 6a may underlie QRS widening in *Mbnl1^ΔE2/ΔE2^* mice. Significantly, QRS widening is known to greatly increase the chance of sudden death in the general human population with cardiomyopathy and in DM1 patients^11^,^12^,^65^. Additionally, Scn5a loss of function mutations are known to cause sick sinus syndrome^66^ where reduction in the flow of sodium ions alters the ability of the SA node to develop and spread electrical signals. Therefore, this splice error may be of significance both with respect to SA node dysfunction and QRS widening in *Mbnl1^ΔE2/ΔE2^* mice. *Mbnl1^ΔE2/ΔE2^* hearts show altered splicing of *Junctate* and reduction in *Junctin* mRNA levels. As aberrant levels of junctin and junctate are known to alter calcium homeostasis and result in arrhythmia and sudden death^67^,^68^,^69^,^70^, it is possible that these splice errors can further enhance the chance of sudden death in *Mbnl1^ΔE2/ΔE2^* mice. Lastly, it is of interest to note that *Ryr2* mutations have been reported to cause polymorphic ventricular tachycardia and that *Cacna1s* missplicing results in aberrant gating of Ca(V)1.1 calcium channel^71^,^72^. Thus alterations in sodium and calcium currents may underlie the conduction defects and sudden death observed in *Mbnl1^ΔE2/ΔE2^* mice

Mbnl1 depletion leads to multi-focal myofibrillar death, calcification and fibrosis prominently in the septum and ventricles (Fig. 5). These structural alterations can act as electrical insulators to result in conduction blocks. Enhanced myocyte death or fibrosis in the His-Purkinje system or ventricle can lead to diminished R wave amplitudes and QTc widening. With regard to the development of fibrosis, it should be noted that whole body inactivation of the splice regulator, RBM20 in mice results in altered titin splicing with the persistence of large, elastic embryonic N2BA titin isoforms in adult mouse hearts^37^. This change increases myocardial compliance and the reduced recoil of flaccid titin filaments is predicted to play a causal role in an adaptive increase in collagen biosynthesis and the development of fibrosis, arrhythmias and sudden death in RBM20 knockout mice^36^,^37^. RNAs encoding the large embryonic N2BA titin isoforms are not observed in adult *Mbnl1^ΔE2/ΔE2^* hearts. In contrast, both in DM1 patient hearts and in *Mbnl1^ΔE2/ΔE2^* hearts, enhanced expression of embryonic myomesin I, an isoform that is more elastic and thus more compliant than the adult isoform of myomesin I is observed^38^,^39^,^40^,^73^. As myomesin is adapted to bear mechanical stress and M-line proteins must be stretched and coiled during muscle contraction and relaxation, increased elasticity of embryonic Myomesin I is predicted to trigger an adaptive increase in fibrosis in a manner similar to that predicted for titin missplicing in RBM20 deficient mice^36^,^37^,^38^,^39^,^40^. We observe that Mbnl1 depletion results in diminished inclusion of *Capn3* exon 16 & 17. Capn3 is a calcium dependent protease that binds to titin and cleaves several cytoskeletal proteins. Absence of Capn3 mediated substrate cleavage has been shown to result in muscle fiber death^41^. Analysis of Capn3 splice variants has shown that *Capn3* exon 16 is required for fodrin cleavage^42^. Thus the diminished inclusion of *Capn3* exon16, is of interest with respect to the cardiac myofiber death observed in *Mbnl1^ΔE2/ΔE2^* hearts.

We observe a very high rate of *Tnnt2* exons 4 and 5 inclusion in *Mbnl1^ΔE2/ΔE2^* mice. This inclusion pattern is more marked than that observed in E18 *Mbnl1^+/+^* hearts (Fig. 7d). The importance of this isoform switching in the development of cardiac hypertrophy, is underscored by an intron 3 polymorphism that alters exon 4 splicing and predisposes to left ventricle hypertrophy^74^. Mutations in Zasp/Cypher in humans are associated with cardiac hypertrophy^75^ and re-expression of embryonic RNA isoforms of *MyomI*, *Asph*, *Mbnl2* have been reported in rat models of cardiac hypertrophy^76^. Lastly, we observe a cascade of splice errors reminiscent of embryonic splice patterns in RNAs regulating cytoskeleton and sarcomere assembly and function including *Trim55, Mapt, Pdlim3, Pdlim5, Sorbs1, Sorbs2, Fhod1* and *Spag9* in *Mbnl1^ΔE2/ΔE2^* hearts. We speculate that the aberrant isoform stoichiometry of these proteins could enhance myocyte fragility, death or maladaptive hypertrophy in *Mbnl1^ΔE2/ΔE2^* hearts. Elucidation of the causal role of these splice errors in DM1 cardiac pathology in future studies should provide important mechanistic insights into DM1 cardiac disease.

Human heart failure is associated with high morbidity and is an end-stage of various forms of heart disease^77^. Several characteristic changes are observed in end-stage failing hearts, with the best documented alterations at the molecular and organ level being a transcriptional and post-transcriptional splicing reprogramming to resemble that of the embryonic heart, various degrees of hypertrophy, mitochondrial dysfunction, alteration of sarcomere and cytoskeleton architecture, aberrant calcium handling, increase in myocyte cell death by apoptosis or necrosis and enhanced extracellular matrix formation^78^. Although the switchback to an embryonic splice program is well established in heart failure its potential role in either accelerating healing or in triggering further dysfunction and death is unclear. For this reason there has been a great interest in identifying and characterizing splice regulators that control embryonic to adult splice transitions. Although RBM20, ASF/SF2 and SC35 were initially regarded as potential candidates that regulate the transition of embryonic to adult splice programs, it is now clear that the MBNL1 and CUG-BP1, which plays an opposing role to MBNL1, play a central role in this phenomenon. Results from this study suggest that re-expression of embryonic isoforms in heart failure may be maladaptive.

It is significant to note that the persistence of the embryonic splice program observed in *Mbnl1^ΔE2/ΔE2^* hearts is reminiscent of that observed in human heart failure. Indeed several of the classic markers for human heart failure including re-expression of embryonic RNA splice isoforms of *Myom I*, *Tnnt2* and low *Junctin* levels characterize the *Mbnl1^ΔE2/ΔE2^* hearts^33^,^70^,^79^,^80^. Interestingly, global analysis of splice changes in human heart failure demonstrates a significant overrepresentation of MBNL motifs in sequences flanking differentially spliced exons^79^. It should be noted that MBNL1 levels increase and CUG-BP1 levels decrease during heart development^60^ and molecular events that change these trajectories can reinstate embryonic splice programs in the adult heart. As alterations in MBNL1 and CUG-BP1 levels or function can result in splice errors, cardiac dysfunction and death, it will be particularly important to understand the molecular events that regulate the activity of this pair of proteins in heart disease. Such studies are predicted to uncover novel therapeutic avenues that diminish the rates of progression to heart failure and death in both DM1 patients and in the general population.

## Methods

### Ethics Statement

All experiments were performed in accordance with the institutional guidelines of both the University of Southern California (USC), and the University of California, Los Angeles (UCLA). The USC protocol was approved by the Institutional Animal Care and Use Committee at the University of Southern California, Los Angeles (Protocol number: 10347). The UCLA protocol (99-028) was approved by the UCLA Office of Animal Research Oversight.

### Statistical analysis

Kaplan-Meyer curves were developed to examine differences in survival between *Mbnl1^+/+^* and *Mbnl1^ΔE2/ΔE2^* mice, testing the difference in survival using the Mantel-Cox (log rank) test and adjusting for *Mbnl1^ΔE2/ΔE2^* mice that were sacrificed. Further modeling examined the potential of a gender difference within the *Mbnl1^ΔE2/ΔE2^* mice. Analyses were performed in SPSS (v.21); a = 0.05.

### Surface ECG Recording

Electrocardiograms were obtained for at least 15 minutes from each mouse either under light isoflurane anesthesia or subsequent to ketamine/xylazine administration (ketamine 80 mg/kg and xylazine 8 mg/kg) by respectively inserting two Pt needle electrodes (Grass Technologies, West Warwick, RI) or two 29 guage needle electrodes (ADInstruments, Colorado Springs, CO) under the skin in the lead II configuration. Isoflurane induced ECG data were amplified (Grass Technologies) and then digitized for analysis with HEM V4.2 software (Notocord Systems, Croissy sur Seine, France). ECG data recorded through ketamine/xylazine administration were amplified using a Bio Amp (ADInstruments) and digitized using Labchart7 software (ADInstruments). Since mouse ECG waveform shapes are different from human, QRS duration values in this study include the Tri (transient re-entry current) wave, as it is part of the ventricular depolarization phase. T waves tend to be negative going in mice. To correct for the variability in the heart rate observed between mouse genotypes, corrected QTc and STc intervals were calculated using Bazett's Formula: QTc = QT/√RR and STc = ST/√RR.

### Ultrasound Echocardiography

Left ventricular (LV) size, mass, wall thickness, ventricular and valve function and blood flow were assessed using methods previously described^81^,^82^. Measures of chamber dimensions [end-diastolic dimension (EDD); end-systolic function (ESD); ventricular septal thickness (VST); posterior wall thickness (PWT)], heart rate, ventricular function [left ventricular % fractional shortening (LV%FS); velocity of circumferential fiber shortening (Vcf); and left ventricular ejection fraction (LVEF)] and the early (E) and atrial (A) diastolic filling (E/A ratio) were obtained from mice lightly anesthetized with isoflurane (1.0–1.5%) to permit physiological levels of function.

### Morphometry & Histology

All morphometry and histology experiments were carried out primarily as described by Jordan and colleagues^82^.

### RNA analysis

Heart tissue was pulverized under liquid nitrogen. Total RNA was prepared using Trizol (Invitrogen, USA) according to the manufacturer's protocol. RNA from E18 heart tissues was obtained from Zyagen Inc. cDNA was synthesized from 5 μg of total RNA using the cDNA synthesis kit (Thermo Scientific., USA). cDNAs were used for splicing assays using primers and PCR conditions described in Supplementary Table S6. The relative band intensities were measured by densitometry analysis using Gene Tool (Syngene Inc., USA). To identify novel splice isoforms, bands were excised and DNA was extracted using gel extraction kits (Qiagen, USA). Extracted DNAs were cloned into pGEM-T Easy Vector (Promega, USA) according to the manufacturer's protocol. The DNA inserts were sequenced using T7 and SP6 promoter sequencing primers (Integrated DNA Technologies Inc, USA). To measure the level of exon 6a inclusion in *Scn5a*, amplified PCR products were visualized on 2% agarose gel followed by digestion with Sac I (NEB, USA) for 2 h at 37°C. The relative band intensities were measured by densitometry analysis using Gene Tool.

### Western blot analysis

Mouse heart tissues were pulverized in liquid nitrogen and whole cell lysates were prepared by homogenization in modified RIPA buffer (Upstate Inc., USA). Protein extracts were subjected to Western blot analysis with anti-Mbnl1 described by Holt and colleagues^83^. Anti-Mbnl2 and anti-Tubulin antibodies were obtained from Santa Cruz Biotechnology Inc. USA.

## Author Contributions

S.R., D.M.D., J.C. and L.C. conceived and designed the experiments; D.M.D., J.C., A.E., S.P., K.P.R., M.C.J. and M.C.F. performed experiments and analyzed the data; S.R., D.M.D. and J.C. wrote the manuscript; S.R., D.M.D., J.C., A.E., S.P., K.P.R., M.C.J., M.C.F. and L.C. discussed and reviewed the manuscript.

## Supplementary Material

Supplementary Information Supplementary info
