# Supplementary material for: Loss of muscleblind-like 1 results in cardiac pathology and persistence of embryonic splice isoforms
Source: Sci Rep. 2015 Mar 12;5:9042. doi: 10.1038/srep09042 (PMC4356957; doi:10.1038/srep09042)
Supplement: Supplementary Information — Supplementary info [file srep09042-s1.pdf]

Loss of muscleblind-like 1 results in cardiac pathology and persistence of embryonic splice isoforms

Donald M. Dixon[1*], *Jongkyu Choi*[1*], *Ayea El-Ghazali*[1,4], *Sun Young Park*[1], *Kenneth P. Roos*[2], *Maria C. Jordan*[2], *Michael C. Fishbein*[3], *Lucio Comai*[4], *Sita Reddy*[1]

[1]Department of Biochemistry and Molecular Biology, University of Southern California, Los Angeles, CA 90033, USA; [2]Department of Physiology, David Geffen School of Medicine at UCLA, Los Angeles, CA 90095, USA; [3]Department of Pathology and Laboratory Medicine, David Geffen School of Medicine at UCLA, Los Angeles, CA 90095, USA; [4]Department of Microbiology and Immunology, University of Southern California, Los Angeles, CA 90033, USA.

* These authors contributed equally to the study.

LEGENDS FOR SUPPLEMENTARY FIGURES AND TABLES

Supplementary Figure S1. a-f. The full-length gels for Fig. 1b-d.

Supplementary Figure S2. Relative weights (g) at 3-4 months and 5-7 months of age, for male and female *Mbnl1*$^{+/+}$ (Male: 3-4 months: n=12 ; 5-7 months: n= 9; Female: 3-4 months n= 8; 5-7 months n= 7) and *Mbnl1*$^{\Delta E2/\ \Delta E2}$ mice (Male: 3-4 months: n=4 ; 5-7 months: n= 5; Female: 3-4 months n= 4; 5-7 months n=11) are shown. p-values were calculated using the Student's t-test with significance set at p≤0.05.

Supplementary Figure S3. *Mbnl1*$^{\Delta E2/\Delta E2}$ mice show QRS, QTc widening and STc shortening at 2 months

a. Electrocardiogram interval values for male (n=3) and female (n=3) *Mbnl1*$^{+/+}$ and male (n=3) and female (n=3) *Mbnl1*$^{\Delta E2/\Delta E2}$ mice at ~2 months of age are shown. Mice underwent light isoflurane anesthesia at an inducing dosage of 3% isoflurane in oxygen, which was reduced to 1-1.5% isoflurane in oxygen when measurements were recorded. As mouse ECG waveform shapes are different from human, QRS duration values in this study include the Tri (transient re-entry current) wave, as it is part of the ventricular depolarization phase. 80-90 beats were analyzed for each mouse. p-values were calculated using the Student's t-test with significance set at p≤0.05. p values $\leq$ 0.05 are indicated in red.

Supplementary Figure S4. ***Mbnl1***$^{\Delta E2/\Delta E2}$ **mice show widened QRS intervals and STc shortening at 3-4 months**

a. Electrocardiogram interval values for male (n=3) and female (n=5) *Mbnl1*$^{+/+}$ and male (n=3) and female (n=5) *Mbnl1*$^{\Delta E2/\Delta E2}$ mice at ~3-4 months of age are shown. Mice underwent light isoflurane anesthesia at an inducing dosage of 3% isoflurane in oxygen, which was reduced to 1-1.5% isoflurane in oxygen when measurements were recorded. As mouse ECG waveform shapes are different from human, QRS duration values in this study include the Tri (transient re-entry current) wave, as it is part of the ventricular depolarization phase. 80-125 beats were analyzed for each mouse. p-values were calculated using the Student's t-test with significance set at $p \leq 0.05$. p values $\leq$ 0.05 are indicated in red.

Supplementary Figure S5. ***Mbnl1***$^{\Delta E2/\Delta E2}$ **mice anesthetized with ketamine/xylazine show prolonged QTc intervals**.

a. Electrocardiogram interval values for male (n=4) and female (n=6) *Mbnl1*$^{+/+}$ and male (n=3) and female (n=5) *Mbnl1*$^{\Delta E2/\Delta E2}$ mice at 6 months of age are shown. Mice underwent anesthesia via an intraperitoneal injection of 80mg/kg ketamine and 8mg/kg xylazine in saline. QRS duration values in this study include the Tri (transient re-entry current) wave, as it is part of the ventricular depolarization phase in mice. As S waves, following the triwaves, were not prominent in 129sv *Mbnl1*$^{+/+}$ and 129sv *Mbnl1*$^{\Delta E2/\Delta E2}$ mice after ketamine/xylazine treatment QRS intervals were not measured. Beats analyzed were male (n=74) and female (n=77) for *Mbnl1*$^{+/+}$ and male (n=31) and female (n=54) for *Mbnl1*$^{\Delta E2/\Delta E2}$ mice. p-values were calculated using the Student's t-test with significance set at $p \leq 0.05$. p-values $\leq$ 0.05 are indicated in red.

Supplementary Figure 6. ***Mbnl1***$^{\Delta E2/\Delta E2}$ **male mice show ventricular hypertrophy.**

a. Ultrasound echocardiographic evaluation at 2 months and 6-7 months of age for male *Mbnl1*$^{+/+}$ (2 months n=3; 6-7 months n=6) and *Mbnl1*$^{\Delta E2/\ \Delta E2}$ (2 months n=3; 6-7 months n=5) mice are shown. p-values were calculated using Student's t-test with significance set at p≤0.05. Values where p≤0.05 are shown in red.

Supplementary Figure S7. Splice events that are not altered in *Mbnl1*$^{\Delta E2/\Delta E2}$ hearts.

Splicing analyses for the indicated genes were performed by RT-PCR in E18 *Mbnl1*$^{+/+}$ hearts and male *Mbnl1*$^{+/+}$ (n=3) and male *Mbnl1*$^{\Delta E2/\ \Delta E2}$ (n=3) hearts at 4 months of age. Exon numbers and expected band sizes are indicated. Exon numbers are annotated based on Refseq from UCSC genome browser (NCBI37/mm9). Alternatively spliced exons are shown as green boxes. Band intensities were quantified by densitometry. Data are standard error of mean (SEM). Uncropped images are shown in Supplementary Fig. S16-17.

Supplementary Figure S8. Analysis of *Junctin* RNA.

a, b. RT-PCR analysis of *Junctin* RNA using primers located in *Asph* exon1a and exon 5a (**a**) and *Asph* exon 1 and exon 5a (**b**) is shown.

Supplementary Figure S9. Novel splice events detected in *Mbnl1*$^{\Delta E2/\Delta E2}$ hearts.

Splicing analyses for the indicated RNAs were performed by RT-PCR (Fig.7). Gel extracted DNA from each band was cloned into pGEM-T Easy vector and sequenced. Exon numbers are indicated. The sequence of each novel exon (green box) is shown.

Supplementary Figure S10. Full-length gels for Fig. 7b.

Supplementary Figure S11. Full-length gels for Fig. 7c.

Supplementary Figure S12. Full-length gels for Fig. 7d.

Supplementary Figure S13. Full-length gels for Fig. 7e.

Supplementary Figure S14. Full-length gels for Fig. 7f.

Supplementary Figure S15. Full-length gels for Fig. 7g.

Supplementary Figure S16. Full-length gels for Supplemental Fig. S7.

Supplementary Figure S17. Full-length gels for Supplemental Fig. S7.

Supplementary Table S1. a, b. EKG values for male (**a**) and female (**b**) $Mbnl1^{+/+}$ and $Mbnl1^{\Delta E2/\Delta E2}$ mice at 2 months of age with isoflurane anesthesia are tabulated. Data are mean and standard deviation (SD). p-values were calculated using the Student's t-test with significance set at $p \leq 0.05$. Values where $p \leq 0.05$ are shown in red.

Supplementary Table S2. a, b. EKG values for male (**a**) and female (**b**) *Mbnl1*$^{+/+}$ and *Mbnl1*$^{\Delta E2/\Delta E2}$ mice at 3-4 months of age with isoflurane anesthesia are tabulated. Data are mean and standard deviation (SD). p-values were calculated using the Student's t-test with significance set at p≤0.05. Values where p≤0.05 are shown in red.

Supplementary Table S3. a, b. EKG values for male (**a**) and female (**b**) *Mbnl1*$^{+/+}$ and *Mbnl1*$^{\Delta E2/\Delta E2}$ mice at 6 months of age with ketamine/xylazine anesthesia are tabulated. Data are mean and standard deviation (SD). p-values were calculated using the Student's t-test with significance set at p≤0.05. Values where p≤0.05 are shown in red.

Supplementary Table S4. a, b. Ultrasound echocardiographic values recorded from male and female *Mbnl1*$^{+/+}$ and *Mbnl1*$^{\Delta E2/\ \Delta E2}$ mice at 2 months of age are shown. Measurements of the left ventricle taken in this study include ventricular septal thickness (VST), end-diastolic dimension (EDD), posterior wall thickness (PWT), end-systolic dimension (ESD), and left ventricle mass (Lvmass). Heart function assessments included aorta ejection time (Ao-ET), left ventricle percent fractional shortening (Lv%FS), velocity of circumferential fiber shortening (Vcf), left ventricle ejection fraction (LvEF) and the E/A ratio. Data are mean and standard deviation (SD). p-values were calculated using the Student's t-test with significance set at p≤0.05. NA: data could not be collected due to the small size of the mice.

Supplementary Table S5. a, b. Ultrasound echocardiographic values recorded from male (**ai**) and female (**bi & bii**) *Mbnl1*$^{+/+}$ and *Mbnl1*$^{\Delta E2/\ \Delta E2}$ mice at 6-7 months of age are shown. Measurements of the left ventricle taken in this study include ventricular septal thickness (VST),

end-diastolic dimension (EDD), posterior wall thickness (PWT), end-systolic dimension (ESD), and left ventricle mass (Lvmass). Heart function assessments included aorta ejection time (Ao-ET), left ventricle percent fractional shortening (Lv%FS), velocity of circumferential fiber shortening (Vcf), left ventricle ejection fraction (LvEF) and the E/A ratio. Data are mean and standard deviation (SD). p-values were calculated using the Student's t-test with significance set at $p \leq 0.05$. Values where $p \leq 0.05$ are shown in red. **bii** shows the values for the two female *Mbnl1*$^{\Delta E2/\Delta E2}$ mice that showed hypertrophy and functional impairment.

Supplementary Table S6: PCR primers and conditions.

Figure S1

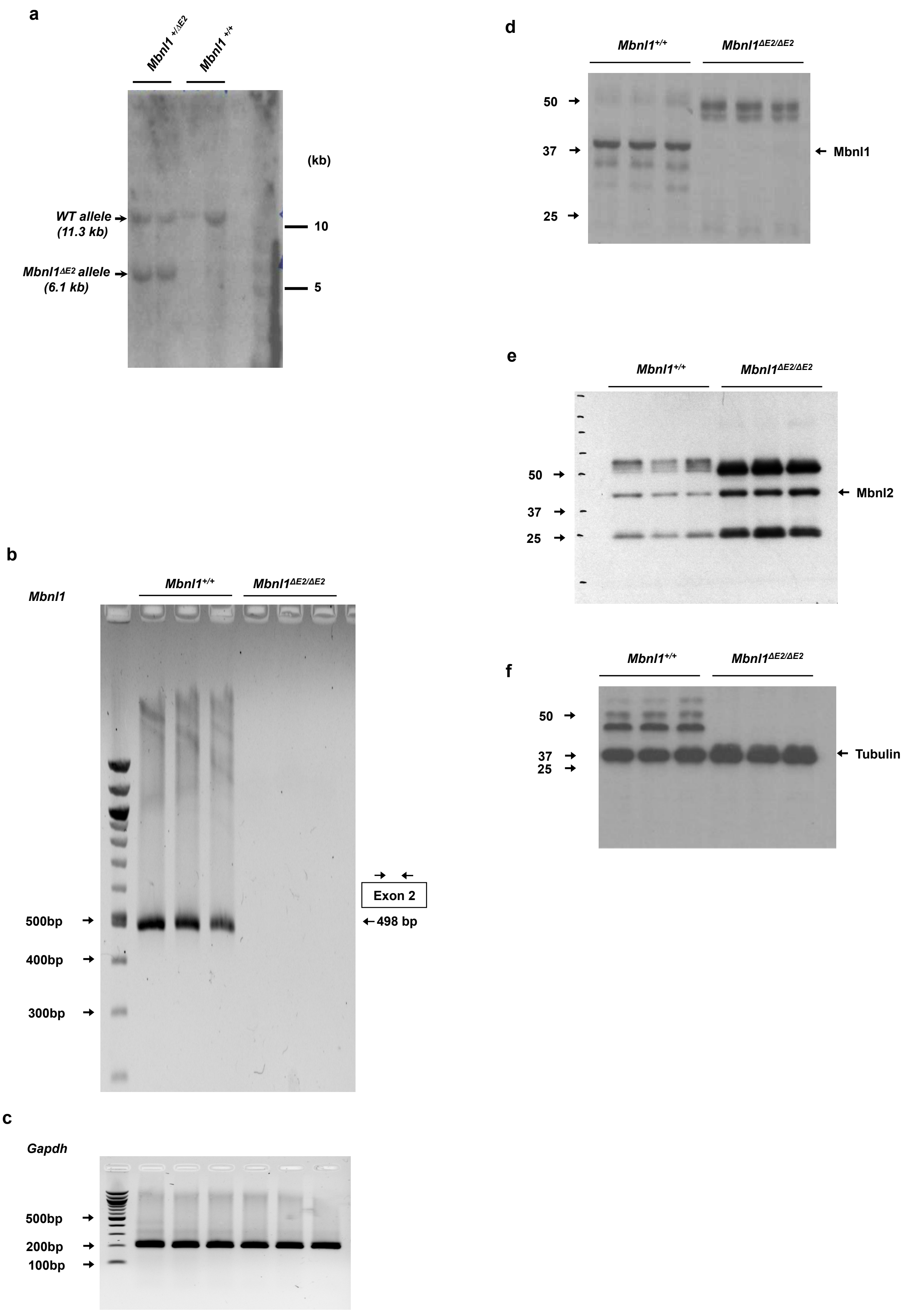

a
Mbnl1$^{+/\Delta E2}$
Mbnl1$^{+/+}$
(kb)
WT allele
(11.3 kb)
Mbnl1$^{\Delta E2}$ allele
(6.1 kb)
10
5
b
Mbnl1
Mbnl1$^{+/+}$
Mbnl1$^{\Delta E2/\Delta E2}$
500bp
400bp
300bp
Exon 2
498 bp
c
Gapdh
500bp
200bp
100bp
d
Mbnl1$^{+/+}$
Mbnl1$^{\Delta E2/\Delta E2}$
50
37
25
Mbnl1
e
Mbnl1$^{+/+}$
Mbnl1$^{\Delta E2/\Delta E2}$
50
37
25
Mbnl2
f
Mbnl1$^{+/+}$
Mbnl1$^{\Delta E2/\Delta E2}$
50
37
25
Tubulin

Figure S2

a

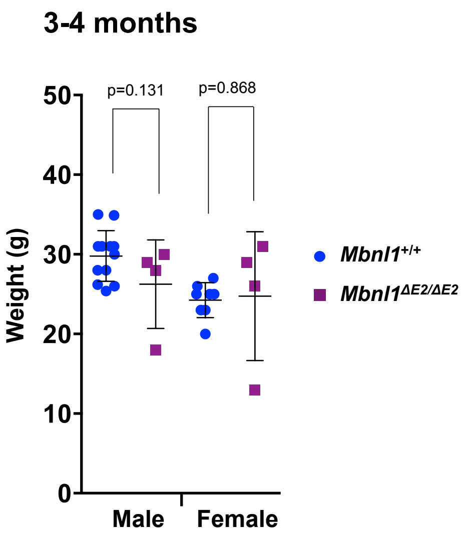
3-4 months
p=0.131
p=0.868
50
40
30
20
10
0
Weight (g)
Male
Female
Mbnl1+/+
Mbnl1ΔE2/ΔE2

b

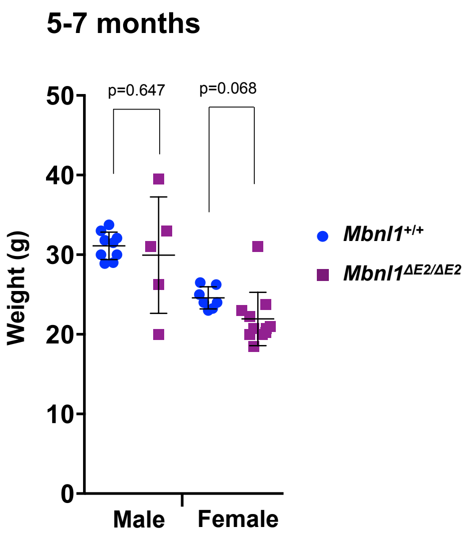
5-7 months
p=0.647
p=0.068
50
40
30
20
10
0
Weight (g)
Male
Female
Mbnl1+/+
Mbnl1ΔE2/ΔE2

Figure S3

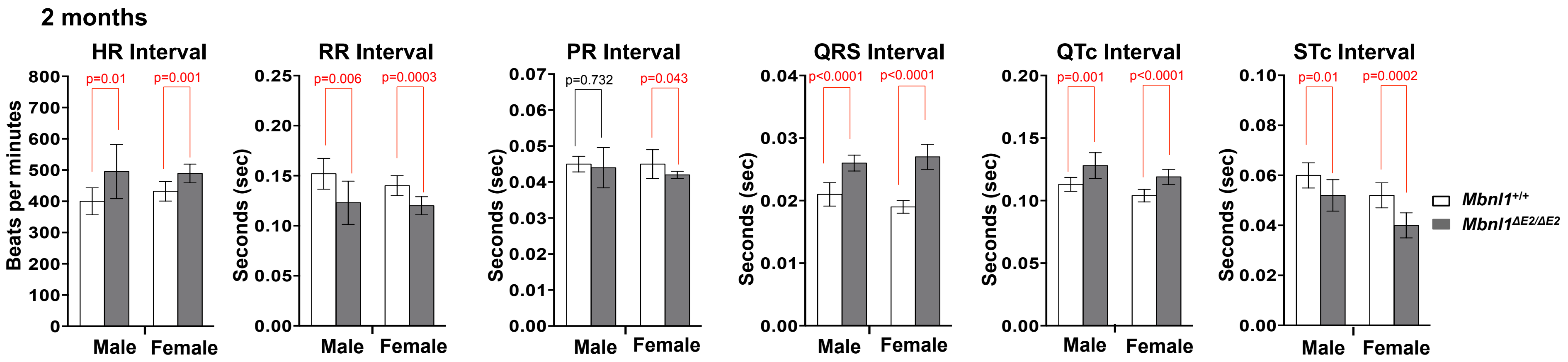
2 months
HR Interval
RR Interval
PR Interval
QRS Interval
QTc Interval
STc Interval
Beats per minutes
Seconds (sec)
p=0.01
p=0.001
p=0.006
p=0.0003
p=0.732
p=0.043
p<0.0001
p<0.0001
p=0.001
p<0.0001
p=0.01
p=0.0002
Male
Female
Mbnl1+/+
Mbnl1ΔE2/ΔE2

Figure S4

3-4 months

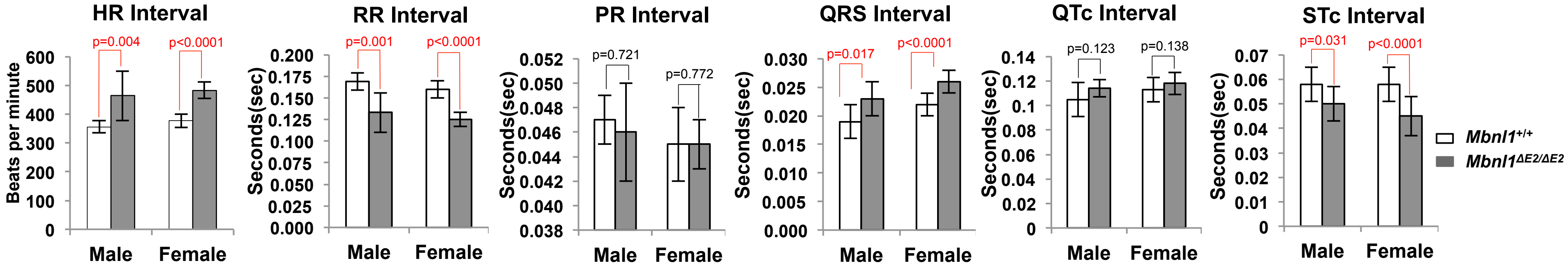

Figure S5

6-7 months

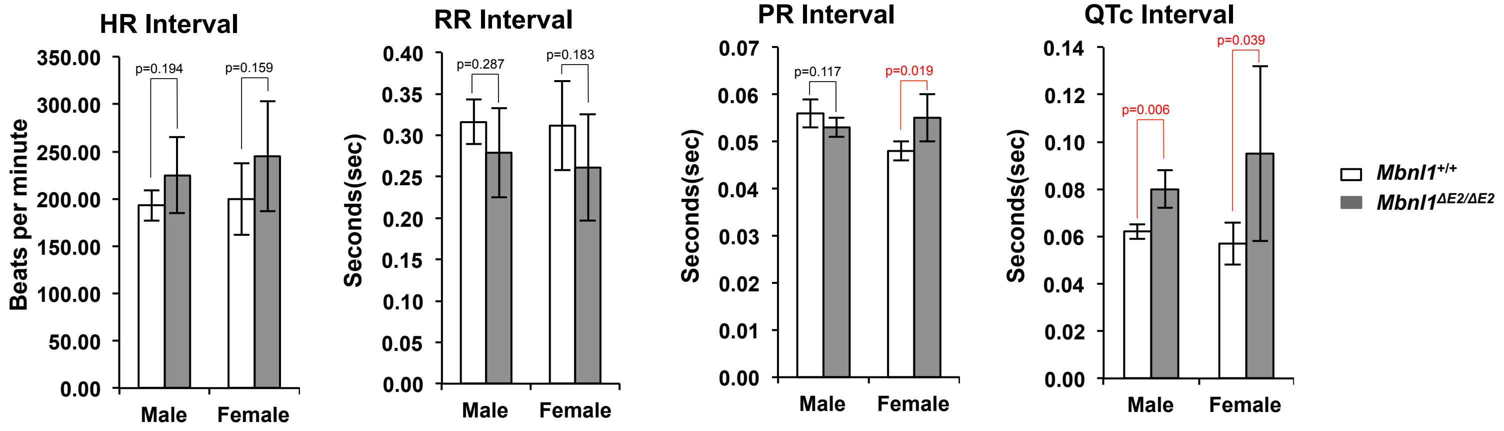

Figure S6

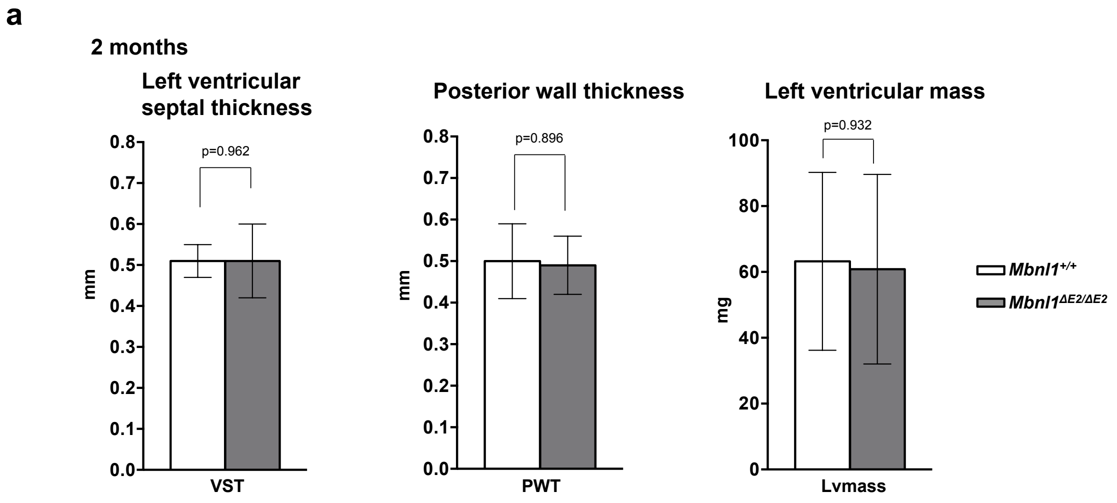
a
2 months
Left ventricular septal thickness
Posterior wall thickness
Left ventricular mass
p=0.962
p=0.896
p=0.932
mm
mg
VST
PWT
Lvmass
Mbnl1+/+
Mbnl1ΔE2/ΔE2

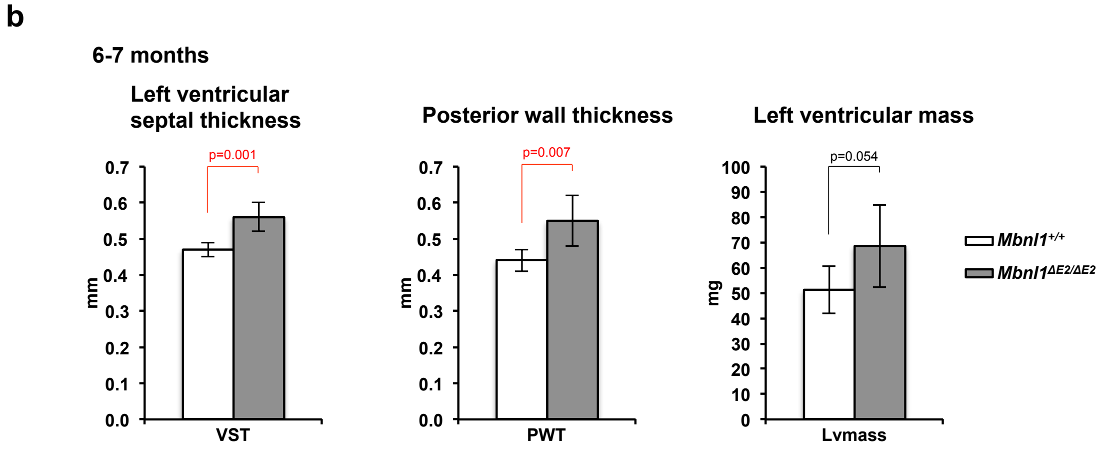
b
6-7 months
Left ventricular septal thickness
Posterior wall thickness
Left ventricular mass
p=0.001
p=0.007
p=0.054
mm
mg
VST
PWT
Lvmass
Mbnl1+/+
Mbnl1ΔE2/ΔE2

Figure S7

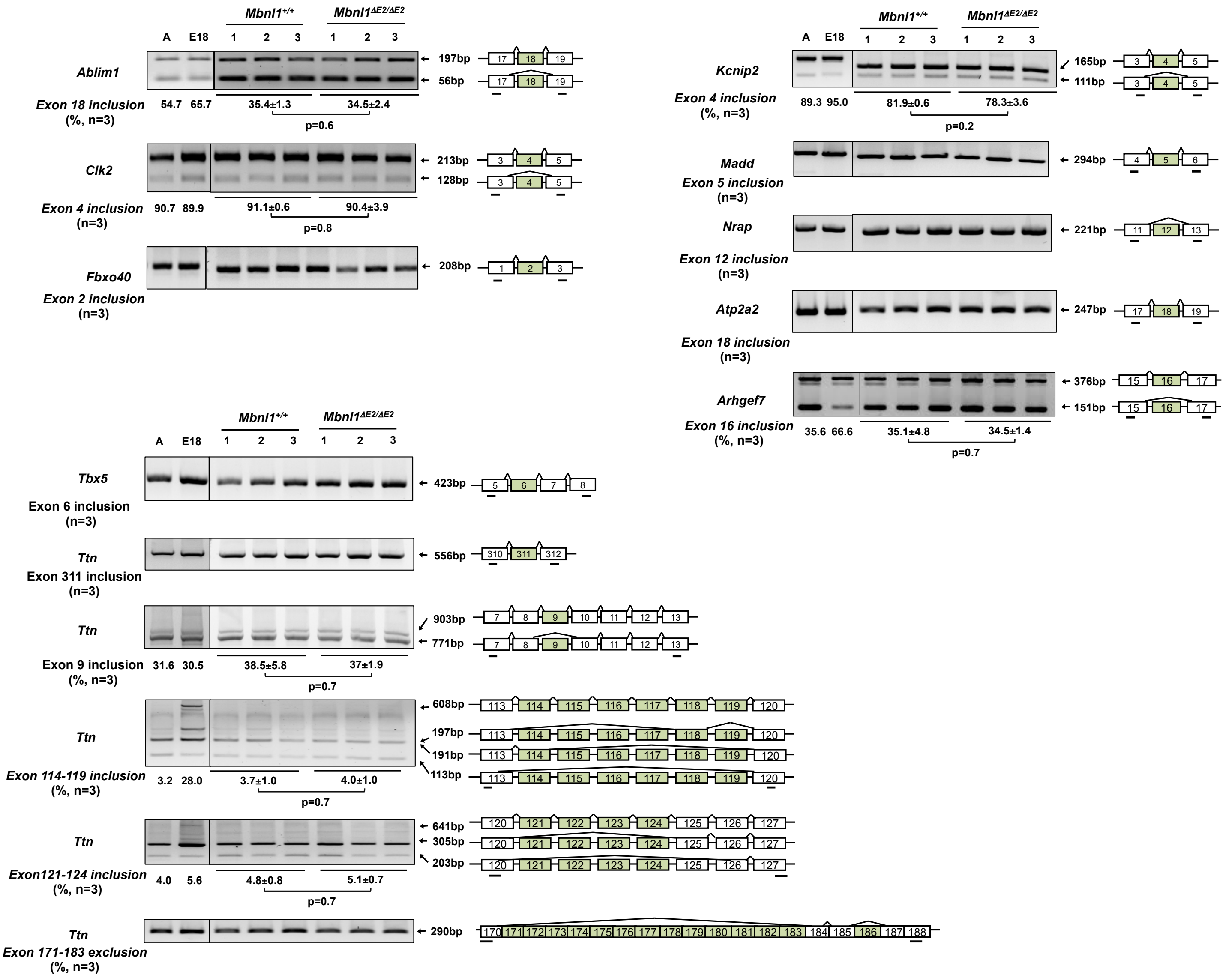

Mbnl1+/+
Mbnl1ΔE2/ΔE2
A
E18
1
2
3
Ablim1
197bp
56bp
Exon 18 inclusion (%, n=3)
54.7
65.7
35.4±1.3
34.5±2.4
p=0.6
Clk2
213bp
128bp
Exon 4 inclusion (n=3)
90.7
89.9
91.1±0.6
90.4±3.9
p=0.8
Fbxo40
208bp
Exon 2 inclusion (n=3)
Kcnip2
165bp
111bp
Exon 4 inclusion (%, n=3)
89.3
95.0
81.9±0.6
78.3±3.6
p=0.2
Madd
294bp
Exon 5 inclusion (n=3)
Nrap
221bp
Exon 12 inclusion (n=3)
Atp2a2
247bp
Exon 18 inclusion (n=3)
Arhgef7
376bp
151bp
Exon 16 inclusion (%, n=3)
35.6
66.6
35.1±4.8
34.5±1.4
p=0.7
Tbx5
423bp
Exon 6 inclusion (n=3)
Ttn
556bp
Exon 311 inclusion (n=3)
Ttn
903bp
771bp
Exon 9 inclusion (%, n=3)
31.6
30.5
38.5±5.8
37±1.9
p=0.7
Ttn
608bp
197bp
191bp
113bp
Exon 114-119 inclusion (%, n=3)
3.2
28.0
3.7±1.0
4.0±1.0
p=0.7
Ttn
641bp
305bp
203bp
Exon121-124 inclusion (%, n=3)
4.0
5.6
4.8±0.8
5.1±0.7
p=0.7
Ttn
290bp
Exon 171-183 exclusion (%, n=3)

Figure S8

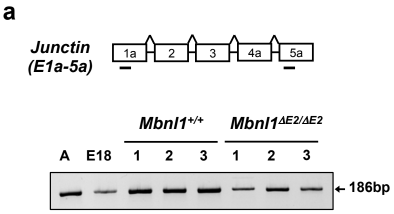
a
Junctin
(E1a-5a)
1a
2
3
4a
5a
Mbnl1+/+
Mbnl1ΔE2/ΔE2
A
E18
1
2
3
1
2
3
186bp

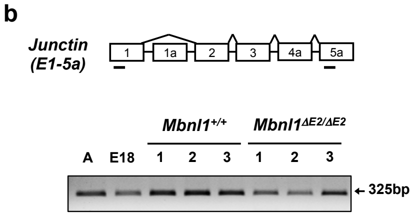
b
Junctin
(E1-5a)
1
1a
2
3
4a
5a
Mbnl1+/+
Mbnl1ΔE2/ΔE2
A
E18
1
2
3
1
2
3
325bp

Figure S9

Myom1 Exon 18a Sequence (188bp)

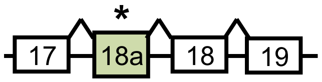

CTTCACATCCCAGTCTCCTCAGGCATTCCCCAGTGTATGAAATTCCATCCTAACATCATGTTCATCCTGGG
ACCTGGCAGGCCGCCCTGCATCCATGAAGTGGTACATCACTGTAACTTGTATCGAACTGTATCTAAAAGC
CATTTCACCTGCAACAGAGCTTGGCTTCTTCCTAACAGCATATTACC

Pdlim3 (_Alp_) Exon 4a Sequence (65bp)

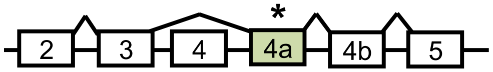

GATGTGAACTACTTTGAACACAAGCACAATATTCGGCCCACACCTTTCATAATTCCAGGCCGAAC

Pdlim3 (_Alp_) Exon 4b Sequence (267bp)

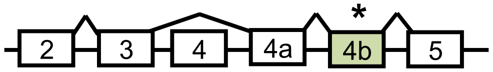

CAGTGGCTGTAGCACTCCTTCCGGTGTTGACTGTGGCAGTGGACGTAGCACCCCTTCTTCCGTCAGTA
CCGTTAGTACAATCTGCCCGGGTGACTTGAAAGTTGCTGCTAAGATGGCCCCTAACATTCCTTTGGAAAT
GGAACTTCCGGACGTGAAGATCGTACACGCTCAGTTTAACACGCCTATGCAGTTGTACTCAGATGACATA
TTATGGAAACACTTCAGGGTCAGGTTGCCACAGCTCTAGGGGAGACATCCTCCATGAG

Figure S10

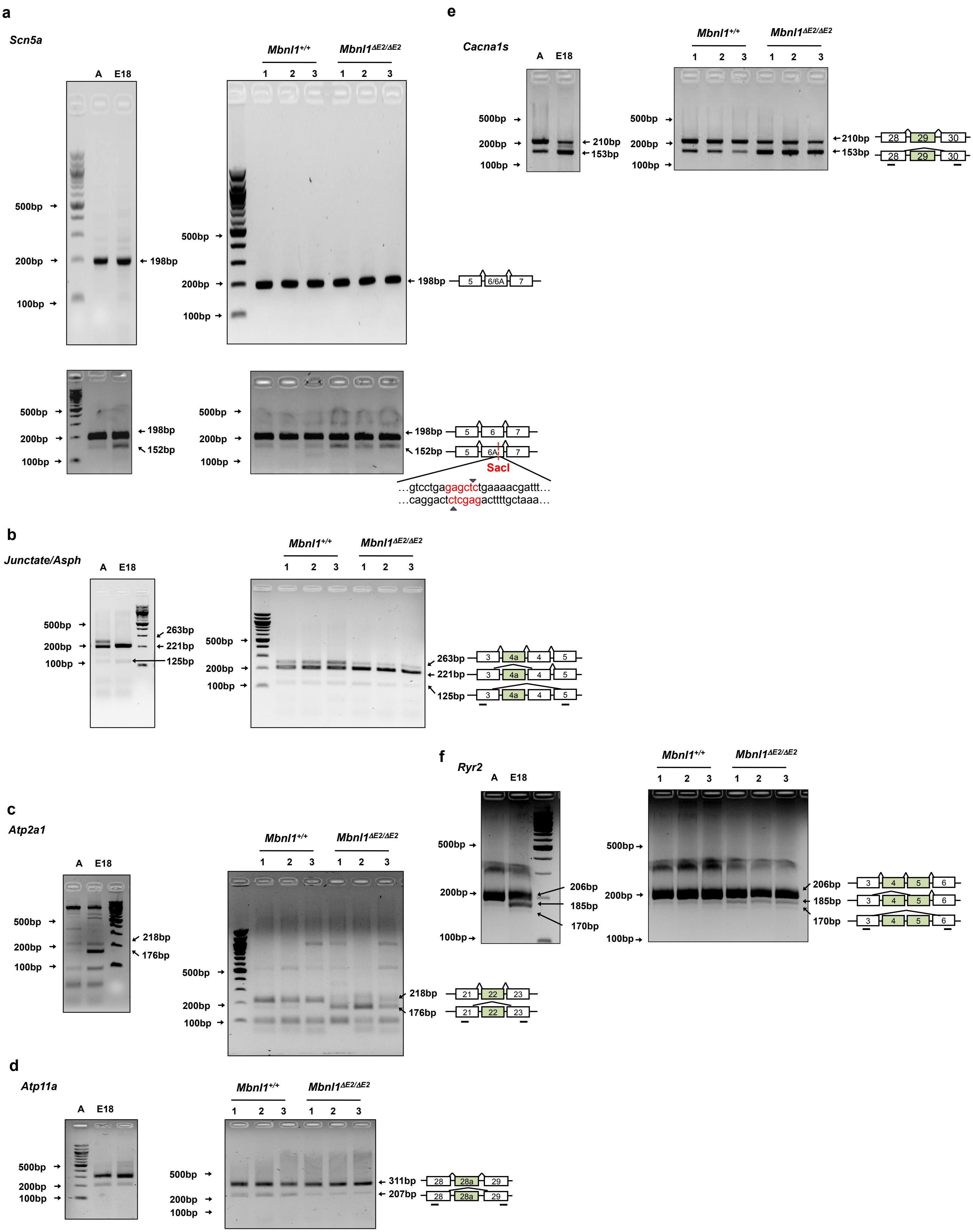

a
Scn5a
A E18
Mbnl1+/+
Mbnl1ΔE2/ΔE2
1 2 3 1 2 3
500bp
200bp
100bp
198bp
152bp
5 6/6A 7
5 6 7
5 6A 7
SacI
…gtcctgagagctctgaaaacgattt…
…caggactctcgagacttttgctaaa…
b
Junctate/Asph
263bp
221bp
125bp
3 4a 4 5
c
Atp2a1
218bp
176bp
21 22 23
d
Atp11a
311bp
207bp
28 28a 29
e
Cacna1s
210bp
153bp
28 29 30
f
Ryr2
206bp
185bp
170bp
3 4 5 6

Figure S11

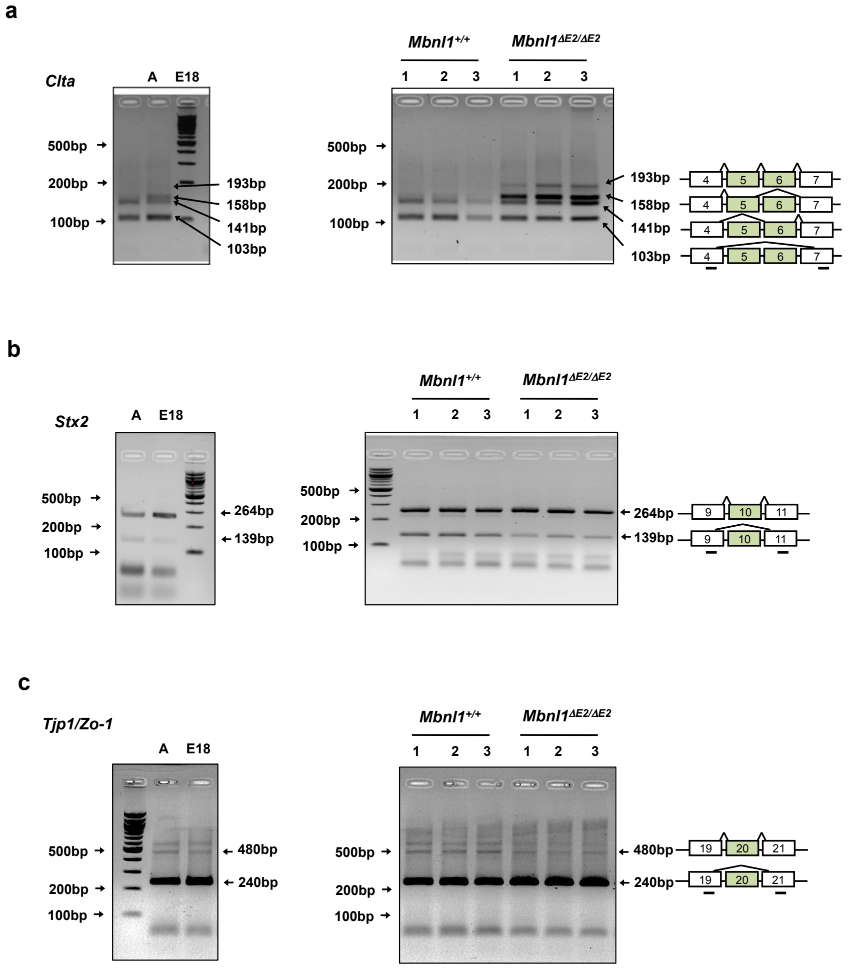
a
Clta
A
E18
Mbnl1+/+
Mbnl1ΔE2/ΔE2
1
2
3
500bp
200bp
100bp
193bp
158bp
141bp
103bp
4
5
6
7
b
Stx2
500bp
200bp
100bp
264bp
139bp
9
10
11
c
Tjp1/Zo-1
480bp
240bp
19
20
21

Figure S12

a

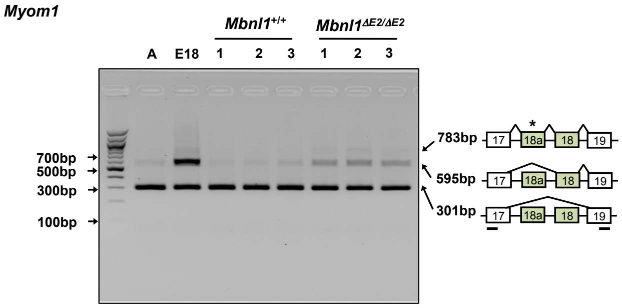

b

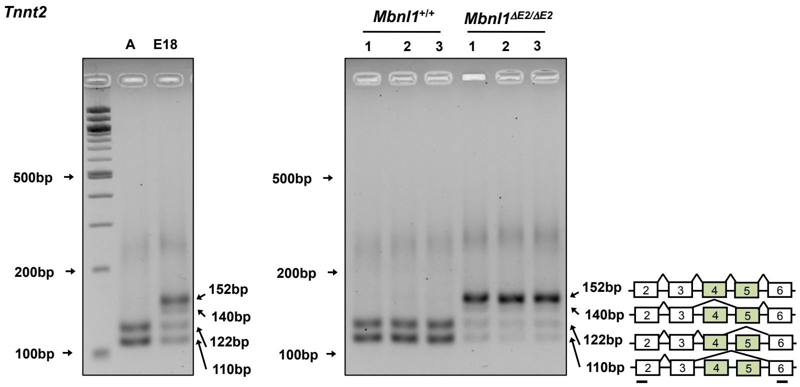

c

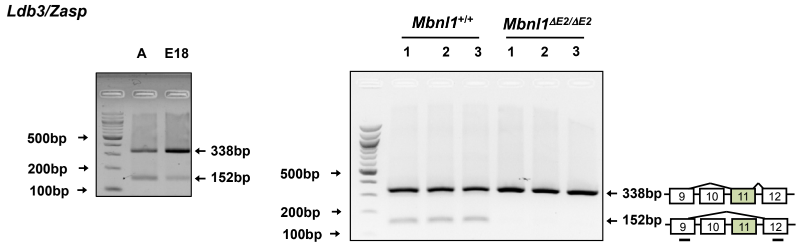

Figure S13

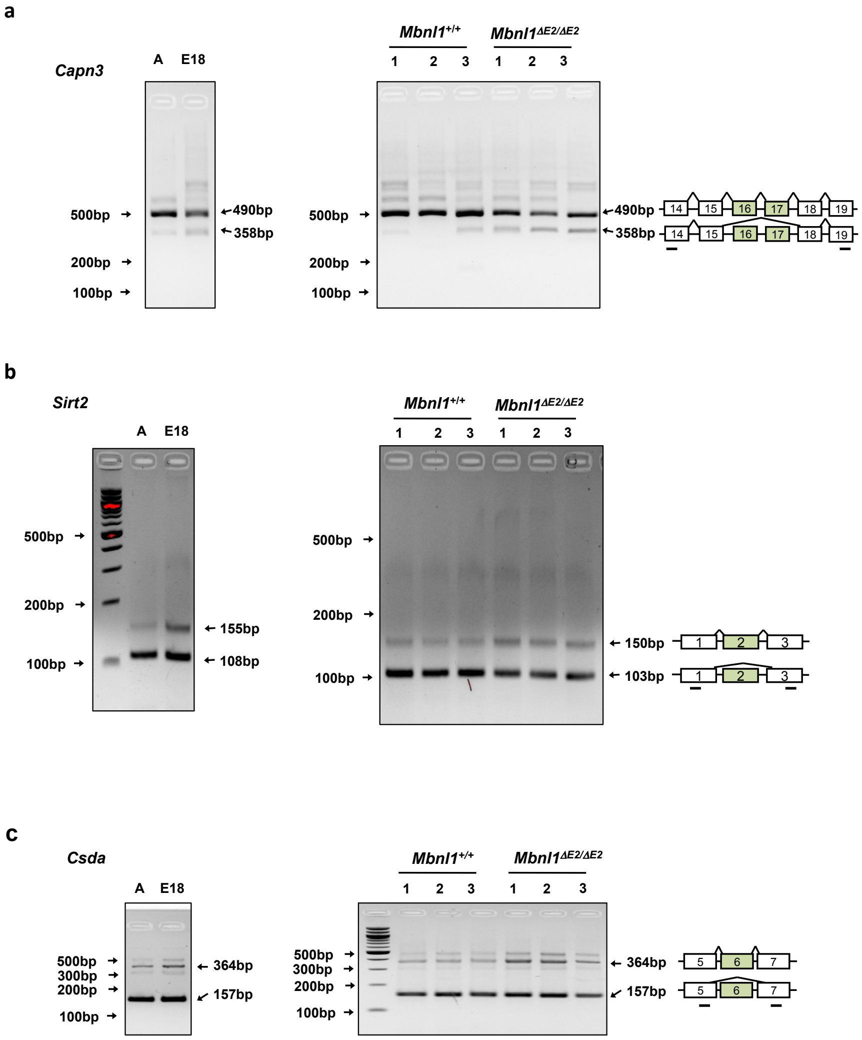
a
Capn3
A
E18
Mbnl1+/+
Mbnl1ΔE2/ΔE2
1
2
3
500bp
200bp
100bp
490bp
358bp
14
15
16
17
18
19
b
Sirt2
155bp
108bp
150bp
103bp
c
Csda
300bp
364bp
157bp
5
6
7

Figure S14

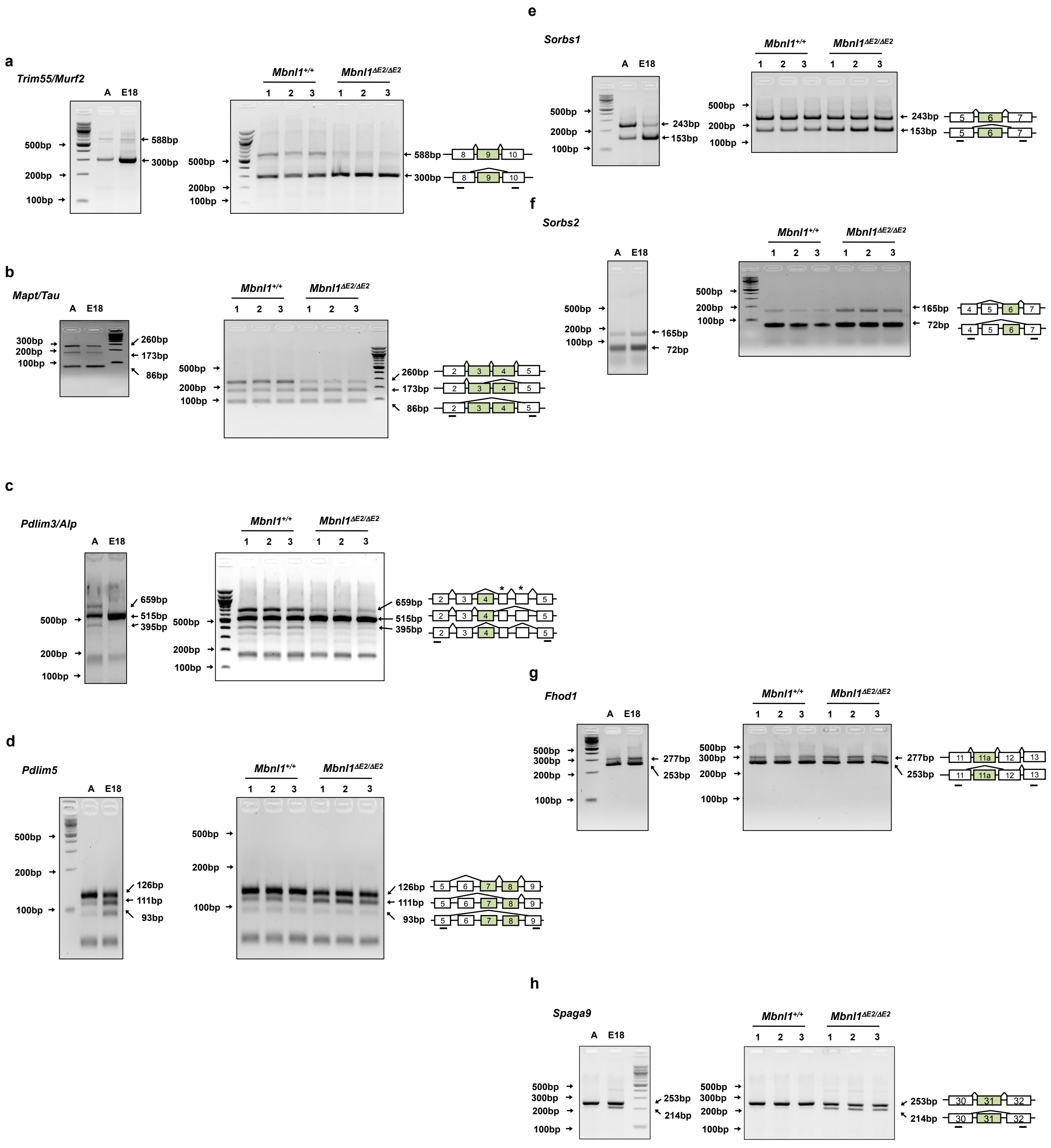
a
Trim55/Murf2
A
E18
500bp
200bp
100bp
588bp
300bp
Mbnl1+/+
Mbnl1ΔE2/ΔE2
1 2 3 1 2 3
8 9 10
b
Mapt/Tau
300bp
200bp
100bp
260bp
173bp
86bp
2 3 4 5
c
Pdlim3/Alp
659bp
515bp
395bp
2 3 4 5
d
Pdlim5
126bp
111bp
93bp
5 6 7 8 9
e
Sorbs1
243bp
153bp
5 6 7
f
Sorbs2
165bp
72bp
4 5 6 7
g
Fhod1
300bp
277bp
253bp
11 11a 12 13
h
Spaga9
253bp
214bp
30 31 32

Figure S15

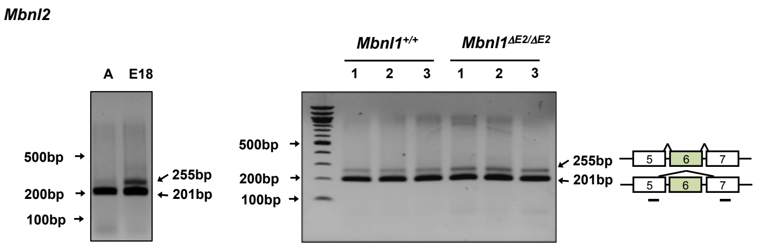
Mbnl2
A
E18
500bp
200bp
100bp
255bp
201bp
Mbnl1+/+
Mbnl1ΔE2/ΔE2
1
2
3
1
2
3
500bp
200bp
100bp
255bp
201bp
5
6
7
5
6
7

Figure S16

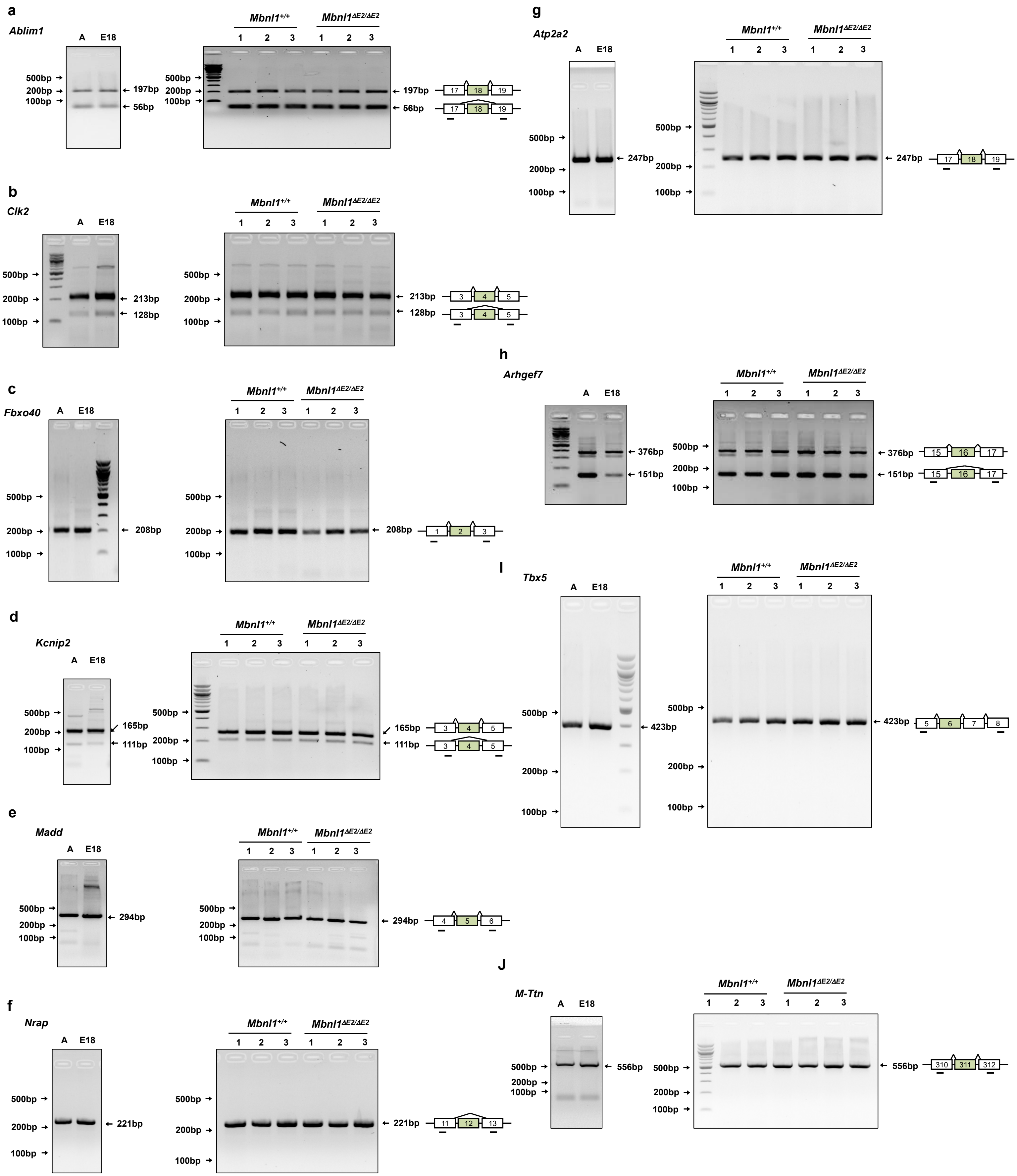

a
Ablim1
A E18
Mbnl1+/+
MbnlΔE2/ΔE2
500bp
200bp
100bp
197bp
56bp
b
Clk2
213bp
128bp
c
Fbxo40
208bp
d
Kcnip2
165bp
111bp
e
Madd
294bp
f
Nrap
221bp
g
Atp2a2
247bp
h
Arhgef7
376bp
151bp
I
Tbx5
423bp
J
M-Ttn
556bp

Figure S17

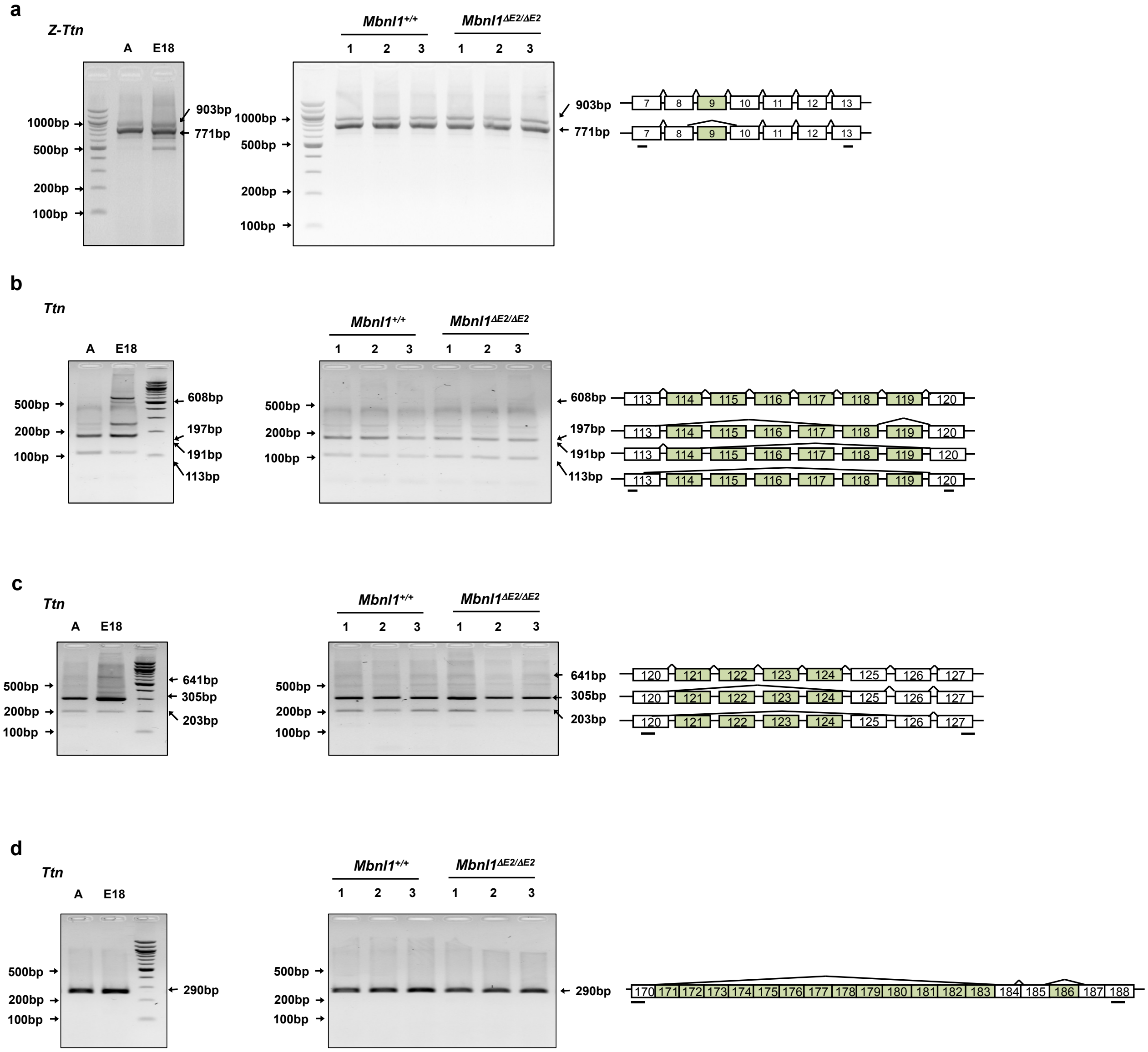
a
Z-Ttn
A
E18
Mbnl1+/+
Mbnl1ΔE2/ΔE2
1
2
3
1000bp
500bp
200bp
100bp
903bp
771bp
b
Ttn
608bp
197bp
191bp
113bp
c
641bp
305bp
203bp
d
290bp

Table S1

a

	Gender	Age (months)	n	# of beats analyzed	Heart Rate (bpm)	RR(sec)	PR(sec)	QRS(sec)	QT(sec)	QTc(sec)	ST(sec)	STc(sec)
Mbnl1$^{+/+}$	Male	2	3	90	400±43	0.152±0.015	0.045±0.002	0.021±0.002	0.044±0.003	0.113±0.006	0.023±0.002	0.060±0.005
Mbnl1$^{\Delta E2/\Delta E2}$	Male	2	3	87	495±87	0.123±0.022	0.044±0.006	0.026±0.001	0.045±0.025	0.128±0.103	0.018±0.031	0.052±0.006
p-value					0.0096	0.0056	0.7319	<0.0001	0.4398	0.0013	0.0007	0.0098

b

	Gender	Age (months)	n	# of beats analyzed	Heart Rate (bpm)	RR(sec)	PR(sec)	QRS(sec)	QT(sec)	QTc(sec)	ST(sec)	STc(sec)
Mbnl1$^{+/+}$	Female	2	3	80	432±31	0.140±0.010	0.045±0.004	0.019±0.001	0.039±0.001	0.104±0.005	0.019±0.002	0.052±0.005
Mbnl1$^{\Delta E2/\Delta E2}$	Female	2	3	87	489±30	0.120±0.009	0.042±0.001	0.027±0.002	0.041±0.002	0.119±0.006	0.014±0.002	0.040±0.005
p-value					0.001	0.0003	0.0434	<0.0001	0.0029	<0.0001	<0.0001	0.0002

Table S2

a

	Gender	Age (months)	n	# of beats analyzed	Heart Rate (bpm)	RR(sec)	PR(sec)	QRS(sec)	QT(sec)	QTc(sec)	ST(sec)	STc(sec)
Mbnl1$^{+/+}$	Male	3-4	3	80	356±21	0.169±0.010	0.047±0.002	0.019±0.003	0.043±0.006	0.105±0.014	0.024±0.003	0.058±0.007
Mbnl1$^{\Delta E2/\Delta E2}$	Male	3-4	3	87	464±86	0.133±0.023	0.046±0.004	0.023±0.003	0.041±0.003	0.114±0.007	0.018±0.002	0.050±0.007
p-value					0.004	0.001	0.721	0.017	0.384	0.123	0.0003	0.031

b

	Gender	Age (months)	n	# of beats analyzed	Heart Rate (bpm)	RR(sec)	PR(sec)	QRS(sec)	QT(sec)	QTc(sec)	ST(sec)	STc(sec)
Mbnl1$^{+/+}$	Female	3-4	5	106	377±23	0.160±0.010	0.045±0.003	0.022±0.002	0.045±0.003	0.113±0.010	0.023±0.003	0.058±0.007
Mbnl1$^{\Delta E2/\Delta E2}$	Female	3-4	5	125	483±29	0.125±0.008	0.045±0.002	0.026±0.002	0.042±0.003	0.118±0.009	0.016±0.003	0.045±0.008
p-value					<0.0001	<0.0001	0.772	<0.0001	0.01	0.138	<0.0001	<0.0001

Table S3

a

	Gender	Age (Months)	n	# of beats analyzed	Heart Rate (bpm)	RR(sec)	PR(sec)	QT(sec)	QTc(sec)
Mbnl1$^{+/+}$	Male	6-7	4	74	193±16	0.316±0.027	0.056±0.003	0.034±0.002	0.062±0.003
Mbnl1$^{\Delta E2/\Delta E2}$	Male	6-7	3	31	225±40	0.279±0.054	0.053±0.002	0.042±0.005	0.080±0.008
p-value					0.194	0.287	0.117	0.059	0.006

b

	Gender	Age (Months)	n	# of beats analyzed	Heart Rate (bpm)	RR(sec)	PR(sec)	QT(sec)	QTc(sec)
Mbnl1$^{+/+}$	Female	6-7	6	77	200±38	0.312±0.054	0.048±0.002	0.032±0.005	0.057±0.009
Mbnl1$^{\Delta E2/\Delta E2}$	Female	6-7	5	54	245±58	0.261±0.064	0.055±0.005	0.047±0.018	0.095±0.037
p-value					0.159	0.183	0.019	0.066	0.039

Table S4

a

	Gender	Age (weeks)	n	Heart Rate	VST (mm)	EDD (mm)	PWT (mm)	ESD (mm)	Ao-ET (ms)	Lv%FS	Vcf	LvEF	Lvmass	E/A
Mbnl1+/+	Male	9.0±0.8	3	498±41	0.51±0.04	3.85±0.64	0.50±0.09	2.75±0.49	52.2±0.07	28.7±1.06	5.50±0.21	63.3±1.13	63.2±27.02	1.49±0.07
Mbnl1 $^{\Delta E2/\Delta E2}$	Male	7.2±2.2	3	473±56	0.51±0.09	3.77±0.57	0.49±0.07	2.77±0.29	53.2±6.80	26.2±4.39	5.04±1.41	59.3±6.39	60.9±28.79	1.72±0.73
**p-value**				0.627	0.962	0.887	0.896	0.964	0.844	0.509	0.693	0.462	0.932	0.700

b

	Gender	Age (weeks)	n	Heart Rate	VST (mm)	EDD (mm)	PWT (mm)	ESD (mm)	Ao-ET (ms)	Lv%FS	Vcf	LvEF	Lvmass	E/A
Mbnl1+/+	Female	8.4±0.0	2	NA	NA	3.25±0.07	NA	2.05±0.07	NA	36.9±3.6	NA	73.0±3.8	NA	1.42±0.03
Mbnl1 $^{\Delta E2/\Delta E2}$	Female	10.6±3.1	2	NA	NA	3.30±0.28	NA	2.25±0.35	NA	32.0±4.9	NA	67.6±9.6	NA	1.63±0.3
**p-value**				NA	NA	0.831	NA	0.515	NA	0.373	NA	0.534	NA	0.460

Table S5

a

i

	Gender	Age (weeks)	n	Heart Rate	VST (mm)	EDD (mm)	PWT (mm)	ESD (mm)	Ao-ET (ms)	Lv%FS	Vcf	LvEF	Lvmass	E/A
Mbnl1+/+	Male	21.3±4.0	6	500±46	0.47±0.02	3.73±0.39	0.44±0.03	2.57±0.31	50.3±1.3	31.3±4.7	6.23±1.02	66.5±6.8	51.2±9.4	1.63±0.15
$Mbnl1^{\Delta E2/\Delta E2}$	Male	22.4±3.7	5	524±19	0.56±0.04	3.82±0.33	0.55±0.07	2.52±0.29	44.0±2.1	33.8±7.4	7.74±1.90	69.32±8.5	68.5±16.3	1.73±0.22
**p-value**				0.321	***0.001**	0.702	***0.007**	0.804	***0.0002**	0.500	0.127	0.557	***0.054**	0.418

b

i

	Gender	Age (weeks)	n	Heart Rate	VST (mm)	EDD (mm)	PWT (mm)	ESD (mm)	Ao-ET (ms)	Lv%FS	Vcf	LvEF	Lvmass	E/A
Mbnl1+/+	Female	28.0±0.3	3	525±43	0.48±0.03	3.63±0.21	0.46±0.02	2.40±0.36	51.5±3.9	34.1±6.8	6.67±1.56	69.7±9.2	50.5±6.2	1.59±0.24
$Mbnl1^{\Delta E2/\Delta E2}$	Female	27.5±0.2	5	499±24	0.50±0.04	4.28±1.10	0.49±0.09	3.26±1.18	51.4±5.9	25.3±9.2	5.12±2.17	56.2±16.7	80.9±42.3	1.96±0.56
**p-value**				0.303	0.634	0.367	0.595	0.276	0.973	0.204	0.327	0.252	0.275	0.345

ii

$Mbnl1^{\Delta E2/\Delta E2}$ **4303**	Female	27.7		511	0.46	5.3	0.46	4.7	54.9	11.3	2.06	29.0	98	NA
$Mbnl1^{\Delta E2/\Delta E2}$ **4304**	Female	27.7		498	0.57	5.6	0.64	4.3	59.6	23.2	3.90	53.2	147	2.59

Tabel S6. PCR primers and conditions:

Primers: Splicing events	PCR Conditions:
**PDZ and LIM domain 3 (*Pdlim3/Alp*)**<br>Forward: 5'- AGC TGC CAA CCT GTG TCC TG -3'<br>Reverse: 5'- GAT CCT GCA GCA CCC TGA AG -3	5 Min at 95°C, [30 sec at 95°C, 30 sec at 60°C, 45 sec at 72°C X35], 10 min at 72°C
**Aspartate beta-hydroxylase (*Asph*)**<br>Forward: 5'- CTA GGA GTC TAT GAT GCG GAT GGC -3'<br>Reverse: 5'- ACT GAT TCC TGA AGA AGG GAC TGA ATT TG -3'	5 Min at 95°C, [30 sec at 95°C, 30 sec at 60°C, 45 sec at 72°C X35], 10 min at 72°C
**ATPase, Ca++ transporting, cardiac muscle, fast twitch 1 *(Atp2a1)***<br>Forward: 5'- GCT CAT GGT CCT CAA GAT CTC AC -3'<br>Reverse: 5'- GGG TCA GTG CCT CAG CTT TG -3	5 Min at 95°C, [30 sec at 95°C, 30 sec at 60°C, 45 sec at 72°C X35], 10 min at 72°C
**Clathrin, light chain A (*Clta*)**<br>Forward: 5'- GGC GAT AAA GGA GCT GGA AGA -3'<br>Reverse: 5'- AAT GTC GTT TAC AAA GGC TTC -3'	5 Min at 95°C, [30 sec at 95°C, 30 sec at 60°C, 45 sec at 72°C X35], 10 min at 72°C
**Cold shock domain protein A/Y-box binding portein 3 (*Csda/Ybx3*)**<br>Forward: 5'- GGT GCT GAA GCA GCA AAC GT -3'<br>Reverse: 5'- GGA CTC CAT CCT TCA TCT CCC C -3'	5 Min at 95°C, [30 sec at 95°C, 30 sec at 60°C, 45 sec at 72°C X35], 10 min at 72°C
**Muscleblind-like protein 1 (*Mbnl1*)**<br>Forward: 5'- GCT GCC CAA TAC CAG GTC AAC -3'<br>Reverse: 5'- TGG TGG GAG AAA TGC TGT ATG C -3'	5 Min at 95°C, [30 sec at 95°C, 30 sec at 60°C, 45 sec at 72°C X35], 10 min at 72°C
**Muscleblind-like protein 2 (*Mbnl2*)**<br>Forward: 5'- ACC GTA ACC GTT TGT ATG GAT TAC -3'<br>Reverse: 5'- CTT TGG TAA GGG ATG AAG AGC AC -3'	5 Min at 95°C, [30 sec at 95°C, 30 sec at 60°C, 45 sec at 72°C X35], 10 min at 72°C

Myomesin 1 *(Myom1*)
Forward: 5’- CAT GGA CTG ACG ACT GCT CAG AGC -3’
Reverse: 5’- CTG CAT CGC TGA CGG CCT TGA TG -3’

5 Min at 95°C, [30 sec at 95°C, 30 sec at 55°C, 45 sec at 72°C X35], 10 min at 72°C

Sorbin and SH3 domain-containing protein 2 (*Sorbs2*)
Forward: 5’- CTG AAG AAG TTA ATA ACA AAC CCT TCA AGG TG -3’
Reverse: 5’- CTG AAT CTG GAG ACT GAG ACT CAC G -3’

5 Min at 95°C, [30 sec at 95°C, 30 sec at 60°C, 45 sec at 72°C X35], 10 min at 72°C

Syntaxin 2 (S*tx2*)
Forward: 5’- GCC AAG GAA GAG ACG AAG AAA GC -3’
Reverse: 5’-GGT ACG GTT GCT ATG ACA ATG CTG -3’

5 Min at 95°C, [30 sec at 95°C, 30 sec at 60°C, 45 sec at 72°C X35], 10 min at 72°C

Microtubule-associated protein tau (*Mapt*)
Forward: 5’- GGA GAT TAC ACT CTG CTC CAA -3’
Reverse: 5’- TTC GGG GTG TCT CCG ATG CCT -3’

5 Min at 95°C, [30 sec at 95°C, 30 sec at 60°C, 45 sec at 72°C X35], 10 min at 72°C

Tight junction protein Zo-1 (*Tjp1/Zo-1*)
Forward: 5’- CTG GAC TTA AGC CAG CCT CTC -3’
Reverse: 5’- TCC GAG AAA ACT GGT CTG TGT -3’

5 Min at 95°C, [30 sec at 95°C, 30 sec at 60°C, 45 sec at 72°C X35], 10 min at 72°C

Troponin T type 2 (*Tnnt2*)
Forward: 5’- GTA CGA GGA ACA GGA AGA AGC -3’
Reverse: 5’- GTC TCA GCC TCA CCC TCA GGC TCA -3’

5 Min at 95°C, [30 sec at 95°C, 30 sec at 60°C, 45 sec at 72°C X35], 10 min at 72°C

Tripartite motif containing 55 (*Trim55/Murf-2*)
Forward: 5’- GGA GAG GAT GCA GTA GAA GTA -3’
Reverse: 5’- CAA CCA GGA GAA GGA GAA GAC -3’

5 Min at 95°C, [30 sec at 95°C, 30 sec at 60°C, 45 sec at 72°C X35], 10 min at 72°C

LIM domain binding 3 (*Ldb3/Zasp*)
Forward: 5’- GCA AGA CCC TGA TGA AGA GGC TCT G -3’
Reverse: 5’- CAG TGG TTA CGA CCC GAG GCT TG -3’

5 Min at 95°C, [30 sec at 95°C, 30 sec at 60°C, 45 sec at 72°C X35], 10 min at 72°C

Sodium channel, voltage-gated, type V, alpha subunit (*Scn5a)*
Forward (Ex5) : 5'- TTC TAG CTC GAG GCT TCT GC -3'
Reverse (Ex7) : 5'- CTC CCA CGA TTG TCT TCA GG -3'

3 Min at 94°C, [30 sec at 94°C, 30 sec at 60°C, 15 sec at 72°C X35], 10 min at 72°C

Forward (Ex4) : 5'- CAT GTG CAC CAT CCT AAC CA -3'
Reverse (Ex6) : 5'- CCC AGG TCC ACA AAT TCA GT -3'
Reverse (Ex6A) : 5'- AAA GTT CGA AGA GCC GAC AA -3'

**SacI* Restriction digestion of Exon6A

Actin biding LIM protein 1 (*Ablim1*)
Forward: 5'- CCG AAA ACC ACC CAT CTA CAA -3'
Reverse: 5'- CAC TTG ATC TGC GCC TCG GG -3'

5 Min at 95°C, [30 sec at 95°C, 30 sec at 60°C, 45 sec at 72°C X35], 10 min at 72°C

Aspartate beta-hydroxylase (*Asph*)
Forward: 5'- AGG AAC AAA TTC AGT CCC TTC TTC AGG -3'
Reverse: 5'- CTG TGT CTT CCA CAT GGT AAG TAT CCT C -3'

5 Min at 95°C, [30 sec at 95°C, 30 sec at 60°C, 45 sec at 72°C X35], 10 min at 72°C

ATPase , class VI, type 11A (Atp11a)
Forward: 5'- CCG TTC CTC AGT TAC CAG AGG AT -3'
Reverse: 5'- CAG AGG GGT GAA TTC TGA AAT GCT G -3'

5 Min at 95°C, [30 sec at 95°C, 30 sec at 60°C, 45 sec at 72°C X35], 10 min at 72°C

Calpain 3 (*Capn3*)
Forward: 5'- CAC GGG AAT AAG CAA CAC CTG -3'
Reverse: 5'- GGA CAT TCT TGA GTT CAT CTG CAC -3'

5 Min at 95°C, [30 sec at 95°C, 30 sec at 60°C, 45 sec at 72°C X35], 10 min at 72°C

CDC-like kinase 2 (*Clk2*)
Forward: 5'- CAG CCG AAG GAA ACA CAG AAG G -3'
Reverse: 5'- CTG CGA TGG TCC ACA CAC TG -3'

5 Min at 95°C, [30 sec at 95°C, 30 sec at 60°C, 45 sec at 72°C X35], 10 min at 72°C

F-box protein 40 (*Fbxo40*)
Forward: 5'- TGA TCA ACG GCA AAG GGC TG -3'
Reverse: 5'- CAG AGC AGG TGG CAG CTT -3'

5 Min at 95°C, [30 sec at 95°C, 30 sec at 60°C, 45 sec at 72°C X35], 10 min at 72°C

Formin homolog 2 domain containing 1 (*Fhod1*)
Forward: 5'- CGC CTA CAA ATC CAG CCT TC -3'
Reverse: 5'- TCC CTT GTG CCT GAT GAT CC -3'

5 Min at 95°C, [30 sec at 95°C, 30 sec at 60°C, 45 sec at 72°C X35], 10 min at 72°C

Kv channel interacting protein 2 *(Kcnip2)*
Forward: 5'- CCC TGC CCT CAG TCA GTG AAA -3'
Reverse: 5'- TGC GTG TGA ACT TGG TTT GTT CC -3'

5 Min at 95°C, [30 sec at 95°C, 30 sec at 60°C, 45 sec at 72°C X35], 10 min at 72°C

MAP-kinase activating death domain (*Madd*)
Forward: 5'- CAT TTG CTC TTC CAG ATC CAT CTC GA -3'
Reverse: 5'- CAG GAA CTC CAA TGA TGT ATG GGG TT -3'

5 Min at 95°C, [30 sec at 95°C, 30 sec at 60°C, 45 sec at 72°C X35], 10 min at 72°C

Nebulin –related anchoring protein (*Nrap*)
Forward: 5'- AAT ACC GGC AGG ACT TCC ATA AG -3'
Reverse: 5'- TAG CCA GGC TGC CAA CTT TG -3'

5 Min at 95°C, [30 sec at 95°C, 30 sec at 60°C, 45 sec at 72°C X35], 10 min at 72°C

PDZ and LIM domain 5 (*Pdlim5*)
Forward: 5'- CAG GGT GAC ATT AAG CAG CAA AAT GG -3'
Reverse: 5'- GGC ATC ACT GTG AGT GGG TAT GTG -3'

5 Min at 95°C, [30 sec at 95°C, 30 sec at 60°C, 45 sec at 72°C X35], 10 min at 72°C

ATPase, Ca++ transporting, cardiac muscle, fast twitch 2 (*Atp2a2*)
Forward: 5'- TGA CGG CGG TCC AAG AGT CT -3'
Reverse: 5'- CAT GGA CAA GCA GAT GGA GC -3'

5 Min at 95°C, [30 sec at 95°C, 30 sec at 60°C, 45 sec at 72°C X35], 10 min at 72°C

Sirtuin 2 (*Sirt2*)
Forward: 5'- CAG AGC AGT CGG TGA CAG TCC -3'
Reverse: 5'- TCT CTG CCT CTC CAC CAG TG -3'

5 Min at 95°C, [30 sec at 95°C, 30 sec at 60°C, 45 sec at 72°C X35], 10 min at 72°C

Sorbin and SH3 domain containing 1 (*Sorbs1*)
Forward: 5'- CTG GCA ACC ATG GAC CAG AC -3'
Reverse: 5'- CTG ACT TGC TTT CAT GCT TCG -3

5 Min at 95°C, [30 sec at 95°C, 30 sec at 60°C, 45 sec at 72°C X35], 10 min at 72°C

T-box 5 (*Tbx5*)
Forward: 5'- CAA ACT CAC CAA CAA CCA CC -3'
Reverse: 5'- GCC AGA GAC ACC ATT CTC AC -3'

5 Min at 95°C, [30 sec at 95°C, 30 sec at 60°C, 45 sec at 72°C X35], 10 min at 72°C

Titin (Ttn* E310-E312*)
Forward: 5'- GTG TGA GTC GCT CCAGAA ACG -3'
Reverse: 5'- CCA CCA CAG GAC CAT GTT ATT TC -3'

5 Min at 95°C, [30 sec at 95°C, 30 sec at 60°C, 45 sec at 72°C X35], 10 min at 72°C

Titin (*Ttn* E7-E13)
Forward: 5’- TGT TGC GAC TGT CGT TGC TG -3’
Reverse: 5’- TCC ACA TGC GTA GGC TCT CTG -3’

5 Min at 95°C, [30 sec at 95°C, 30 sec at 60°C, 45 sec at 72°C X35], 10 min at 72°C

Titin (*Ttn* E113-120)
Forward: 5’- CAG CCA AAG TGC CTG AAA AG -3’
Reverse: 5’- AGC GGC TTC CTT CTT CTT TG -3’

5 Min at 95°C, [30 sec at 95°C, 30 sec at 60°C, 45 sec at 72°C X35], 10 min at 72°C

Titin (*Ttn* E120-127)
Forward: 5’- GCT CCC GAA GAA AAG ATT CC -3’
Reverse: 5’-GGT GGA GGC TCC TCA ATT TT -3’

5 Min at 95°C, [30 sec at 95°C, 30 sec at 60°C, 45 sec at 72°C X35], 10 min at 72°C

Titin (*Ttn* E170-188)
Forward: 5’- AAG ACA GCT GTT CCC CAA AA -3’
Reverse: 5’-TTC TTC CAC AGC CTC CTC TG -3’

5 Min at 95°C, [30 sec at 95°C, 30 sec at 60°C, 45 sec at 72°C X35], 10 min at 72°C

***Junctin* (E1a-5a)**
Forward: 5’- TGC ATG GAT TGA AGA AAT CAA -3’
Reverse: 5’- CAC AGC TTT CCC TCT CTC CTT -3’

5 Min at 95°C, [30 sec at 95°C, 30 sec at 60°C, 45 sec at 72°C X30], 10 min at 72°C

***Junctin* (E1-5a)**
Forward: 5’- TAG CAG CGG TAG CAG CAG TA -3’
Reverse: 5’- CAG CTC TTC TTT GAT GGG TTC -3’

5 Min at 95°C, [30 sec at 95°C, 30 sec at 60°C, 45 sec at 72°C X30], 10 min at 72°C

Ryanodine receptor 2 (*Ryr2*)
Forward: 5’- CGG ACC TGT CTA CTG CAC CTT TGT -3’
Reverse: 5’- CAT ACC ACT GTA GGA ATG GCG TAG CA -3’

5 Min at 95°C, [30 sec at 95°C, 30 sec at 60°C, 45 sec at 72°C X30], 10 min at 72°C

Voltage-dependent L-type calcium subunit (*Cacna1s*)
Forward: 5’- GAGATC CTT GGA ATG TGT TTG ACT TCC T -3’
Reverse: 5’- GGT TCA GCA GCT TGA CCA GTC TCA T -3’

5 Min at 95°C, [30 sec at 95°C, 30 sec at 60°C, 45 sec at 72°C X30], 10 min at 72°C

Rho guanine nucleotide exchange factor 7 isoform (*Arhgef7*)
Forward: 5’- GCA GAC GAA GGT CAC ATC TGT GAG C -3’
Reverse: 5’- CAC AGC GAA CTC CTC GTC CGA AG -3’

5 Min at 95°C, [30 sec at 95°C, 30 sec at 60°C, 45 sec at 72°C X30], 10 min at 72°C

**C-jun-amino-terminal kinase-interacting protein (*Spag9*)**<br>Forward: 5’- GGA CTG GAA ATG GTGTCATTA TCT CCA T -3’<br>Reverse: 5’-ACT GCC ACA AAG AAT TTC ACA G -3’	5 Min at 95°C, [30 sec at 95°C, 30 sec at 60°C, 45 sec at 72°C X30], 10 min at 72°C
**Glyceraldehyde-3-phosphate dehydrogenase (*Gapdh*)**<br>Forward: 5'-AGA GAC GGC CGC CGC ATC TTC TTG TG-3’<br>Reverse: 5’-TCT GGG TCC CAG TGA TGG CAT GG-3’	5 Min at 95°C, [30 sec at 95°C, 30 sec at 60°C, 45 sec at 72°C X30], 10 min at 72°C
